# Test-retest reliability of dynamic functional connectivity parameters for a two-state model

**DOI:** 10.1162/netn_a_00437

**Published:** 2025-03-20

**Authors:** Xiaojing Fang, Michael Marxen

**Affiliations:** Department of Psychiatry, Technische Universität Dresden, Dresden, Germany

**Keywords:** Neuroimaging, fMRI, Connectomics, Brain states, Reproducibility, Sliding window analysis

## Abstract

Reliability of imaging parameters is of pivotal importance for further correlation analyses. Here, we investigated the test-retest reliability of two dynamic functional connectivity (dFC) brain states and related parameters for different scan length, atlases with 116 versus 442 regions, and data centering in 23 participants and reproduced the findings in 501 subjects of the Human Connectome Project. Results showed an integrated and a segregated brain state with high intraclass correlation coefficient (ICC) values of the states between sessions (0.67 ≥ ICC ≥ 0.99). The most reliable dFC parameter was state prevalence with an ICC ≈ 0.5 for ∼15 min of uncentered data, while other parameters, such as mean dwell time, were much less reliable. While shorter scans and within-subject data centering further reduce reliability, the atlas choice had no effects. Spearman’s correlations among dFC parameters strongly depend on data centering. The effect of global signal regression and a higher number of states is discussed. In conclusion, we recommend formulating hypotheses on cross-sectional differences and correlations between dFC measures of brain integration and other subject-specific measures in terms of state prevalence, especially in small-scale studies.

## Author Summary

We investigated the reliability of popular parameters characterizing dynamic brain states in two datasets. We found high reliability of clustering results with a two-state model but only medium to low reliability of the parameters, of which state prevalence across different strategies was the most reliable.

## INTRODUCTION

The analysis of functional connectivity (FC), measuring interregional synchrony of spontaneous brain activity (Friston, 1994), is a key method in neuroscience (Friston, 2011). While early studies assumed that FC was stationary, its dynamic nature (Chang & Glover, 2010) is now recognized (Hutchison et al., 2013). Nevertheless, dynamic FC (dFC) metrics intended for cross-sectional difference and correlation analyses need to be reliable, that is, trait-like. A lot of findings revealed not only neural correlates of resting-state dFC with electroencephalography power (Chang et al., 2013; Tagliazucchi et al., 2012; Thompson et al., 2013) but also associations of the connectivity with demographics, consciousness, behavior, cognition (Hutchison et al., 2013; Preti et al., 2017; Shine et al., 2016), and various neurological and psychiatric disorders, such as Parkinson’s disease (Zoller et al., 2019) and schizophrenia (Calhoun et al., 2014; Hutchison et al., 2013).

Many methods have been used to investigate temporal variations of connectivity, such as time-frequency analysis (Chang & Glover, 2010), time-series modeling (Eavani et al., 2013; Lindquist et al., 2014), change-point detection (Cribben et al., 2013), and coactivation pattern analysis (Liu & Duyn, 2013). Here, we focus on sliding window analysis (SWA), possibly the most popular approach (Allen et al., 2014; Choe et al., 2017; Hutchison et al., 2013; Preti et al., 2017), which divides a time course into small windows and computes FC measures for each window (Chang & Glover, 2010). The test-retest reliability of dFC metrics is a major concern for further cross-sectional analysis (Calhoun et al., 2014; Handwerker et al., 2012; Hlinka & Hadrava, 2015), especially as reliability is bound to decrease with a smaller window size and a shorter scan time due to the low signal-to-noise ratio of resting-state functional magnetic resonance imaging (rs-fMRI) signals, artifact signals from non-neural factors (e.g., respiration, cardiac pulsation and noise) (Hutchison et al., 2013), and fluctuations of the measures on the time scale of hours and days (e.g., circadian effects) (Orban et al., 2020). Higher reliability provides higher power to detect potential cross-sectional effects and is, therefore, a quality measure for subject-specific variables.

Surprisingly, only a few reports of test-retest reliability for brain states were published. One study revealed partial reliability for five states and derived parameters (i.e., duration and transitions) (Z. Yang et al., 2014). Another study (Van Essen et al., 2013) revealed four k-means coactivation clusters across sessions with intermediate reliabilities of intraclass correlation coefficient (ICC) ≤ 0.54 for temporal fraction (i.e., prevalence [Prev]) or mean dwell time (MDT) (Smith et al., 2018). Choe and colleagues (2017) reported similar values for two k-means connectivity clusters (e.g., ICC = 0.56 for dwell time using SWA).

Although, we do not presume that there is an a priori “correct” number of brain states, when maximizing reliability is the prerogative, choosing *k* = 2 clusters is advantageous for three reasons: (a) reliability of the state centroid connectivity matrices will be better than for larger *k* because the states will be most distinct (i.e., furthest away from each other in the clustering space); (b) for parameters such as MDT, noise reduction through averaging should increase with the total time spent in each state (i.e., [scan time]/*k*, which is largest for *k* = 2); and (c) two states are straightforward to interpret as one more integrated and less segregated state and another less integrated and more segregated state in line with the destinction made by Shine et al. (2016) and utilizing the fundamental concepts of brain organization integration and segregation (Friston, 2009).

This study aims to quantify the test-retest reliability of dFC parameters and to investigate the effect of common processing choices. Naturally, the reliability of dFC parameters is affected by additional factors, such as scan length (Hindriks et al., 2016) and other details of the processing pipelines. Consequently, after validating the interpretation of the two states for our data based on the global graph metrics modularity and global efficiency, we will address the following questions using a two-state model and a straightforward SWA approach: (a) How reliable are the two states between scanning sessions? (b) How reliable are particular parameters that quantify the dynamics (see the Reliability of dFC parameters section) for this model? (c) What is the effect of scan length on the reliability? (d) What is the effect of choosing an atlas with a larger number of regions? (e) What is the effect of within-subject centering of the connectivity matrices before clustering, which will remove differences between subjects in static connectivity? On the basis of studying these questions in imaging data of 23 subjects scanned at our own imaging facility as well as in 501 subjects from a public dataset, we will make recommendations regarding the utility of these dFC parameters for identifying between-subject differences in brain function. We will, furthermore, discuss the effect of global signal regression (GSR) (Power et al., 2017) and increasing the number of brain states (Abrol et al., 2017).

## METHODS

These methods have been described in a similar form for different participants in the preregistration for “Linking Need for Cognition to a Two-State Model of Dynamic Brain Connectivity during Rest” (osf.io/286fb) and in the preprint of this paper (Fang & Marxen, 2022).

### Participants, Data Acquisition, and Preprocessing

We initially conducted this study in a small subject sample (our own data) with an in-house imaging protocol acquired at our local scanner and published our findings as a preprint (Fang & Marxen, 2022). See Supporting Information Methods Section S1.1.1. Due to the small sample size, however, confidence intervals were very large. Thus, we repeated our analyses in a larger cohort of subjects taken from the Human Connectome Project (HCP) (Van Essen et al., 2013), which employs a similar fMRI acquisition sequence. See Supporting Information Methods Section S1.1.2. The preprocessing and utilized atlases are described in Supporting Information Methods Section S1.1.3.

### Extraction of Functional Brain States

#### SWA.

In the current study, an instance of one dFC brain state is defined as a connectivity matrix of Fisher z-values based on Pearson’s correlation that was computed for the time window of 40 × repetition time (TR) = 39.48 s based on a previous study (Zhang et al., 2018) for OWN data and 55 × TR = 39.6 s for HCP data. The SWA was computed in DynamicBC toolbox (Liao et al., 2014) (github.com/guorongwu/DynamicBC) with a step size of 1 TR resulting in 961 brain-state instances (i.e., dFC matrices) per scanning run for OWN data and in 1,146 instances for HCP data. Time series were extracted by spatially averaging all voxel-wise signals within each region of interest (ROI).

#### Clustering of state instances and back-projection.

The two-state (i.e., state I and S) classification of the dFC matrices was performed in MATLAB 2018b (MathWorks Inc., Natick, MA, USA) by assigning each matrix to one of *k* = 2 clusters in k-means clustering (Lloyd, 1982). Cosine distance (CD) was employed in clustering to measure the similarity between each state instance and the centroids of a cluster, that is, CD = 1 − *cos* (*α*), where *α* is the angle between two vectorized dFC matrices. Thus, CD ∈[0, 2], with 0 being the smallest distance, that is, the highest similarity (Karahanoğlu & Van De Ville, 2015). Moreover, *k* = 2 was found to be optimal in OWN data according to silhouette statistics (Rousseeuw, 1987) computed for cluster numbers *k* = 2 … 20 (see Supporting Information Figure S1). For session B of OWN data, dFC matrices were assigned to state I or S by back-projecting each matrix onto the cluster centroids from session A (i.e., brain state instances from Session B that were assigned to the state/cluster with the smaller CD to the corresponding cluster centroid of session A). Cluster centroids were defined here as the group mean of the normalized (i.e., divided by the root of the sum of squares of all matrix elements), dFC matrices for the respective state in each session. Distances between all state instances and the two cluster centroids were plotted to validate the clustering procedure. For HCP data, we divided the 501 subjects into three groups equally (167 subjects for each) for computational reasons: We clustered the session A of one of the three groups (A1) into states I and S and back-projected the remaining five session/groups (A2, A3, B1, B2, and B3) onto the resulting centroids. To avoid temporal sequence effects, we randomly labeled the first session in 83 subjects and the second scanning session in the remaining 84 subjects as “Session A,” similar to the procedure in OWN data.

##### Processing pipeline variations.

We investigated 2 × 2 × 2 = 8 different processing pipelines or strategies. Three factors were varied:

1. The length of the rs-fMRI time series: 1,000 frames (16’27”) versus 500 frames (8’14”) for OWN data and 1,200 frames (14’24”) versus 600 frames (7’12”) for HCP data. For the analysis with fewer frames, we only considered the first half of the frames of a run.
2. The brain atlas used to define the ROIs (see below): 116 ROIs versus 442 ROIs.
3. Within-subject centering of dFC matrices versus “uncentered”: For the centered data, the within-subject-within-session mean connectivity matrix was subtracted from the dFC matrices before the clustering, which eliminates differences in static FC between subjects. As the resulting cluster centroids no longer reflect FC strength in this case, we added session-level mean connectivity matrices back to the centroid when appropriate. When comparing mean state matrices without centering, we subtracted the mean of the compared centroids because, otherwise, even not-matching states show high correlations.

#### dFC parameters.

We computed seven different dFC parameters of the brain based on the state sequence of each subject in each session:

1. MDT: the mean time one participant maintained state I or S continuously during a run. Note that we excluded all windows within the very first and last states from the computation of MDT since the beginning/end of the state is unknown. Consequently, only individual dFC series with at least two state transitions were used to calculate MDT for at least one of the states.
2. Prev: the fraction of windows spent in state I or S with respect to the total number of windows in the dFC series. Note: *Prev*_*I*_ = 1 − *Prev*_*S*_.
3. Intertransition interval (ITI): the average time length remaining in any state before transitioning to the other state during scans, that is, *ITI* = (*MDT*_*I*_ + *MDT*_*S*_)/2 for an even number of states in a series. Note that only dFC series including at least three state transitions were considered for the computation.
4. State variability (Var): the mean Euclidean distance of each state instance of one subject to the run average of all state instances within the same state:

${Var}_{I,S}=\frac{1}{T_{I,S}}\sum_{t=1}^{T_{I,S}}D_{\text{Euclid}}\left(C_{t},\frac{1}{T_{I,S}}\sum_{t´=1}^{T_{I,S}}\left(C_{t´}\right)\right)\;$, where *C*_*t*_ is the dFC matrix at time window *t* and *D*_*Euclid*_(*C*_*a*_, *C*_*b*_) is the Euclidean distance between the vectorized dFC matrices *C*_*a*_ and *C*_*b*_. Note that this definition does not use CD or the above definition of the cluster centroid because CD is not invariant to translations in the clustering space.

Following the above definitions, the seven parameters are not completely independent. Nevertheless, each of them represents a different aspect of brain state dynamics and may maximize associations with other parameters. Note that six of seven parameters are specific to each state. The number of subjects used to compute each parameter is given in Supporting Information Table S4.

### Data Analyses

#### Optimal number of clusters by silhouette statistics.

We computed silhouette statistics (Rousseeuw, 1987) for the OWN data as a measure of the optimal number of clusters.

#### Reliability of cluster centroids.

To evaluate the reliability of cluster centroids between sessions, we computed CD and the ICC (Bartko, 1966) between the vectorized FC matrices, a popular measurement that quantifies test-retest reliability and was previously used in other rs-fMRI studies (Choe et al., 2017; Zhang et al., 2018; Zuo & Xing, 2014):

$ICC=\frac{\sigma_{s}^{2}}{\sigma_{s}^{2}+\sigma_{e}^{2}}\approx \frac{{MS}_{bs}-{MS}_{ws}}{{MS}_{bs}+\left(k-1\right)\times {MS}_{ws}}$, where $\sigma_{s}^{2}$ indicates the between-subject variance and $\sigma_{e}^{2}$ the within-subject variance observed in the data. The original equation was estimated by sample mean squares from analysis of variance (Bartko, 1966) in this study, where *MS*_*bs*_ is the mean square between subjects, *MS*_*ws*_ is mean square within subjects, and *k* = 2 is the number of measurements. Thus, ICC values may become less than 0 in this framework if variability within the subjects is greater than between subjects (i.e., “divergence” within a group). As we observed that not-matching states are also highly correlated, we also report ICC and CD after subtracting the mean of the compared cluster centroids, which strongly reduces the similarity of not-matching states.

#### Graph-theoretical analyses of cluster centroids.

To describe the functional characteristics of the two brain states (i.e., cluster centroids), we computed the two global graph parameters, modularity and global efficiency, based on weighted connectivity (Cohen & D'Esposito, 2016; Rubinov & Sporns, 2010) for the 16’27” OWN data in brain connectivity toolbox (Version 2019-03-03, nitrc.org/frs/?group_id=241) using Fisher *z* = 0 as a connection threshold:

1. Modularity $Q=\sum_{i=1}^{m}\left(e_{ii}-{\left(\sum_{j=1}^{m}e_{ij}\right)}^{2}\right)$, where *e*_*ij*_ means the proportion of edges that link a node in module *i* to any node in module *j*, and *m* the number of all nonoverlapping modules generated by Newman’s modularity algorithm (Newman & Girvan, 2004).
2. Global efficiency $E_{\text{global}}=\frac{1}{n\left(n-1\right)}\sum_{i\neq j}{d_{ij}}^{-1}$, where *n* is the number of nodes, and *d*_*ij*_ the minimum distance between node *i* and *j*. In this study, we only computed the distance based on connectivity between the nodes with positive strength (Barch et al., 2013).

We evaluated the 95% confidence intervals within these graph measures by generating 1,000 bootstrapping samples of the dFC matrices for each state/cluster of session A to evaluate the replicability of these measures in session B.

#### Reliability of dFC parameters.

We computed values of ICC and Spearman’s correlation to quantify the reliability of the dFC parameters between the two sessions. Confidence intervals of the reliability were estimated by bootstrapping with 1,000 samples. We are also providing *p* values for both parameters.

#### Correlations between dFC parameters.

To quantify the interdependence between the dFC parameters, we calculated Spearman’s correlations between subjects using the averaged parameter values across the two sessions.

#### Effects of GSR.

During preprocessing, a regressor for the global brain signal was included in addition to the mean white matter, cerebrospinal fluid signals, and motion parameters. This analysis, as well as the analysis described in the following Comparison with a five-state model section, was conducted for 16 min of uncentered HCP data with the smaller atlas only. By using the methods from the Reliability of cluster centroids section, we investigated the reliability of the brain states based on the preprocessed data with and without GSR. In addition, following the Graph-theoretical analyses of cluster centroids section, we studied the reliability of the dFC parameters with and without GSR.

#### Comparison with a five-state model.

In addition to *k* = 2 during the clustering procedure, *k* = 5 was used. The effect of two versus five states was studied for two scenarios within subjects: (a) clustering in session A1 (167 subjects) and back-projecting in the paired session B1 (notation “A1 ➔ B1”) and (b) clustering separately in each session A1 and B1 (notation “A1 versus B1”). For each approach, we investigated the reliability of the cluster centroids and the reliability of the dFC parameters. Our hypothesis was that reliabilities would be higher for back-projection than for repeated clustering and higher for the two-state model than for the five-state model.

## RESULTS

### Optimal Number of Clusters by Silhouette Statistics

Although we chose *k* = 2 clusters out of theoretical considerations, we still evaluated whether our choice was optimal in terms of silhouette statistic in session A. Results are given for OWN data for *k* = 2 … 20 for the different processing strategies in Supporting Information Figure S1 and demonstrate that *k* = 2 results in the highest silhouette score.

### Reliability of Cluster Centroids

#### OWN data.

**Figure 1.**
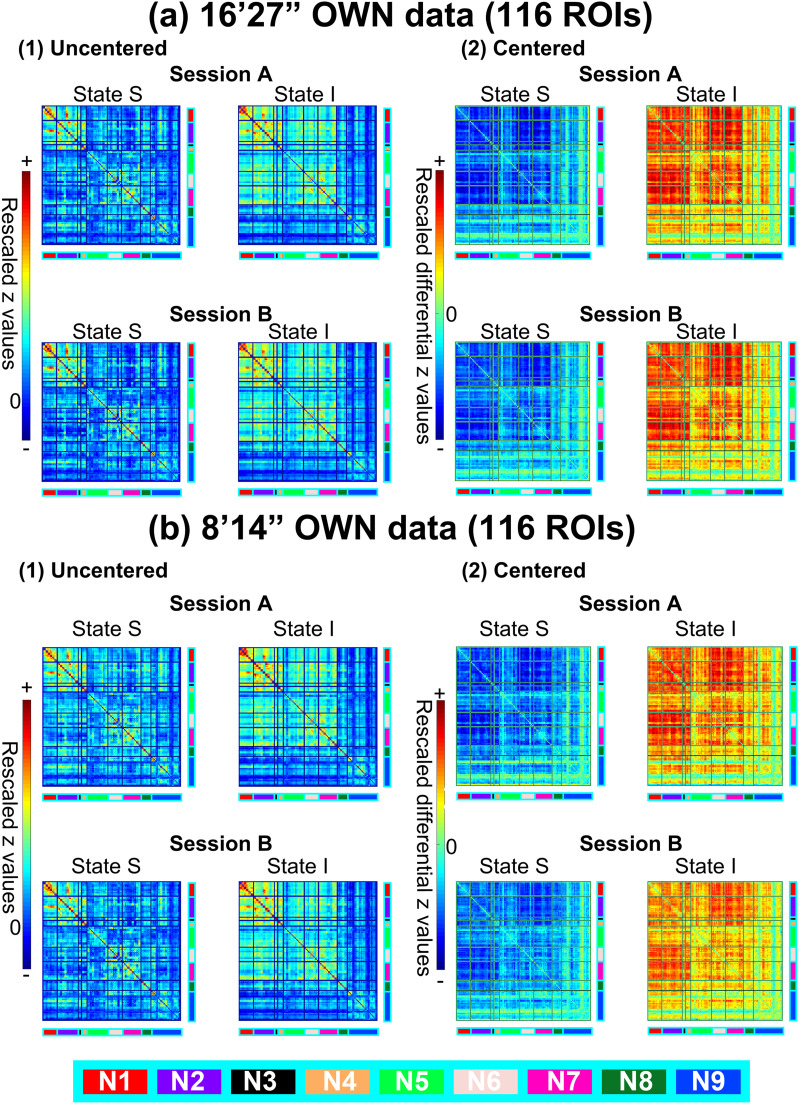
Cluster centroids (*k* = 2) of the dFC matrices based on the Automated Anatomical Labeling (AAL) atlas for different time-length and uncentered/centered OWN data. For session A, k-means clustering was used for the state assignment, while back-projection results are shown for session B. Displayed are the cluster centroids (see the Methods section; N1: visual network; N2: sensory-motor network; N3: dorsal attention network; N4: ventral attention network; N5: limbic network; N6: frontoparietal network; N7: default mode network; N8: basal ganglia network; N9: cerebellum network).

Results of the clustering procedure (session A) and the back-projection (session B), that is, cluster centroids for all processing strategies in OWN data, are shown in Figure 1 for 116 ROIs and Supporting Information Figure S2 for 442 ROIs. The results consistently demonstrate that the dFC matrices cluster around an overall strongly connected (i.e., integrated) state I and a weakly connected (i.e., segregated) state S. For the centered data (Figure 1A2 and 1B2), the centroid of state I showed primarily correlations above the run-mean while state S showed correlations below the run-mean. Supporting Information Figure S3 demonstrates the effect of centering on the computation of CD for state instances of either class. Basically, all instances of state I are moved into the positive quadrant of the clustering space, while the instances of state S are moved into the negative quadrant with a symmetric distribution of the states around 0. In consequence, the CD to the centroid of the other class after centering was always above one and below one for the centroid of the same class and would sum to approximately two. Quantitative reliability between the clusters from session A and B in terms of ICC and CD are given in Table 1 (ICC) and Supporting Information Table S5 (CD), respectively. Note that, for the uncentered data, reliabilities were also computed after removing the mean of the compared state centroids (Tables 1C and 1F, Supporting Information Tables S5C and S5F) to eliminate the bias that uncentered centroids generally exhibited high positive ICC values and low CD values even between different states (Tables 1A and 1D, Supporting Information Tables S5A and S5D). Overall, the reliabilities between corresponding states were generally medium to high (ICC ≥ 0.67), especially for the longer scan time (ICC ≥ 0.86), while not-corresponding states, for example, state S of session A with state I of session B, showed negative ICC values in the centered data. The CD between corresponding states was generally very low (*CD* ≤ 0.37) and very high for not-corresponding states (*CD* ≥ 1.81) after removing the mean of the state centroids in the uncentered data. State reliability generally decreased for shorter scan lengths, more ROIs, and centering.

**Table 1.** Reliability (measured by ICC) between the two clusters for the two sessions

		**16’27” OWN data**		**14’24” HCP data**		**8’14” OWN data**		**7’12” HCP data**	
**116 ROIs**									
**(A) Clusters based on uncentered data**									
		**Ses A**		**Ses A**		**Ses A**		**Ses A**	
		**S**	**I**	**S**	**I**	**S**	**I**	**S**	**I**
**Ses B**	**S**	0.97	0.72	1.00	0.50	0.94	0.79	1.00	0.68
	**I**	0.68	0.99	0.63	1.00	0.70	0.97	0.67	1.00
**(B) Clusters based on centered data**									
		**Ses A**		**Ses A**		**Ses A**		**Ses A**	
		**S**	**I**	**S**	**I**	**S**	**I**	**S**	**I**
**Ses B**	**S**	0.93	−0.14	0.98	−0.09	0.86	−0.13	0.95	−0.10
	**I**	−0.13	0.94	−0.09	0.98	−0.12	0.84	−0.10	0.95
**(C) Clusters based on uncentered data & removing mean of cluster centroids**									
		**Ses A**		**Ses A**		**Ses A**		**Ses A**	
		**S**	**I**	**S**	**I**	**S**	**I**	**S**	I
**Ses B**	**S**	0.78	−0.59	0.94	−0.15	0.60	−0.77	0.96	−0.20
	**I**	−0.50	0.89	−0.14	0.95	−0.63	0.74	−0.20	0.97
**442 ROIs**									
**(D) Clusters based on uncentered data**									
		**Ses A**		**Ses A**		**Ses A**		**Ses A**	
		**S**	**I**	**S**	**I**	**S**	**I**	**S**	**I**
**Ses B**	**S**	0.96	0.57	1.00	0.62	0.93	0.64	1.00	0.66
	**I**	0.55	0.98	0.60	1.00	0.59	0.96	0.64	1.00
**(E) Clusters based on centered data**									
		**Ses A**		**Ses A**		**Ses A**		**Ses A**	
		**S**	**I**	**S**	**I**	**S**	**I**	**S**	**I**
**Ses B**	**S**	0.86	−0.07	0.96	−0.07	0.73	−0.07	0.91	−0.08
	**I**	−0.06	0.87	−0.07	0.96	−0.06	0.67	−0.08	0.91
**(F) Clusters based on uncentered data & removing mean of cluster centroid**									
		**Ses A**		**Ses A**		**Ses A**		**Ses A**	
		**S**	**I**	**S**	**I**	**S**	**I**	**S**	**I**
**Ses B**	**S**	0.67	−0.25	0.97	−0.15	0.39	−0.29	0.97	−0.21
	**I**	−0.24	0.85	−0.14	0.97	−0.22	0.69	−0.21	0.98

Ses: session; S: state S; I: state I.

#### HCP data.

Clustering results in HCP data were consistent with the results in OWN data (Supporting Information Figure S4). State reliabilities in terms of ICC and CD are provided in Table 1 and Supporting Information Table S5. Similar to the results in OWN data, there were high ICC values between the clusters across different strategies (ICC ≥ 0.91) especially for the longer scan time (ICC ≥ 0.94) and negative for not-corresponding states in the centered data (Tables 1A, 1B, 1D, and 1E). In addition, there were very low CD for corresponding states (CD ≤ 0.01) and high for not-corresponding states (CD ≥ 1.98) in the centered data (Supporting Information Tables S5B and S5E). For uncentered data after removing the mean of the state centroids (see Tables 1C, 1F, Supporting Information Tables S5C and S5F), ICC values were above 0.94 for matching states and CD below 0.01. For not-matching states, ICC values were again all negative, CD ≥ 1.99. The decrease in reliability with shorter scan length, more ROIs, and centering was less pronounced in HCP data. This could be due to larger random effects in the small OWN data, that is, the decrease was overestimated by chance.

### Graph-Theoretical Analyses

**Figure 2.**
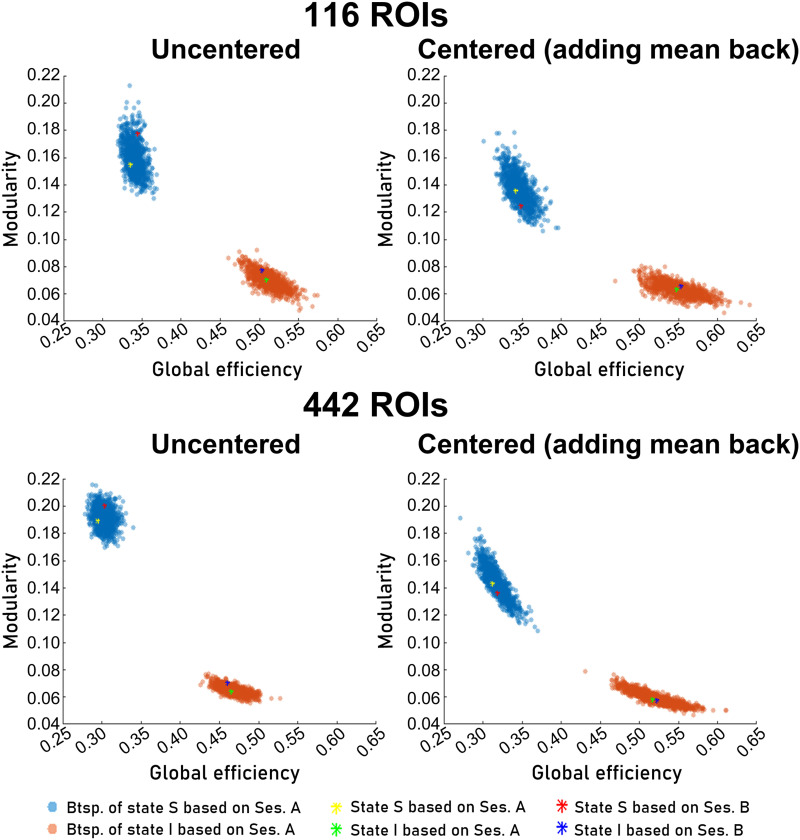
Global graph-theoretical measures of cluster centroids for 16’27” OWN data. Each light blue (brown) point represents the mean FC matrix of state S (I) in session A of 1 of 1,000 bootstrapping samples pulled from *N* = 23 subjects. This point cloud gives an impression of the variability of the modularity and global efficiency of the FC graph of the state centroid. To demonstrate the test-retest consistency of these measures, the values for session B centroids are also shown in yellow, green, red, and dark blue. For centered data, the session mean was added back. Note that session B centroids were computed as mean FC matrices of the respective state instances after back-projection (see the Methods section; Btsp.: bootstrapping; Ses.: session).

To illustrate the nature of the two state centroids, the two global graph parameters modularity and efficiency are plotted in Figure 2 for the 16’27” OWN data including results of the bootstrapping procedure. The results demonstrate that the graphs of the two states are distinct with respect to these two parameters: The state with low connectivity, that is, the “segregated” state (S), had low global efficiency and high modularity, while the state with strong connectivity, that is, the “integrated” (I) state, had high global efficiency and low modularity. Bootstrapping for session A showed that these clusters did not overlap within this two-parameter space. The modularity and global efficiency values of the cluster centroids in session B were in the range of the bootstrapping results for session A (Figure 2 and Supporting Information Table S6), that is, these parameters are reproducible across sessions. Note that individual centering reduced modularity and increased global efficiency for both states (Supporting Information Table S6). Additionally, the two clouds moved closer to each other for the centered data (Figure 2).

### Reliability of dFC Parameters

**Figure 3.**
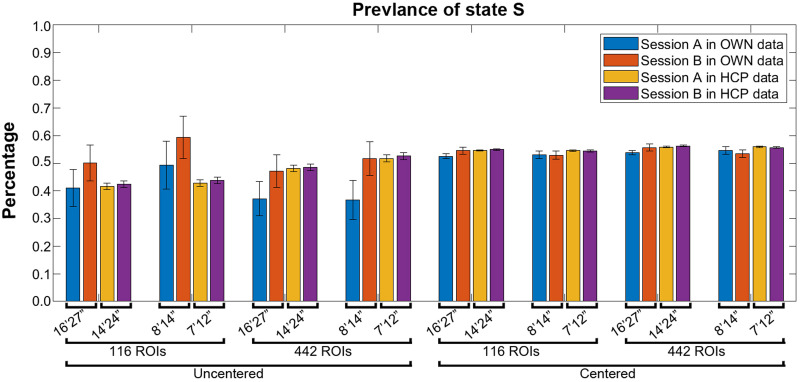
Bar plots of prevalence in sessions for different pipelines and datasets (with standard error of mean).

ICCs and Spearman’s correlations including *p* values and confidence intervals based on bootstrapping over subjects for all parameters and pipelines are given in Table 2 and Supporting Information Table S7. Mean values with standard error of means are given in Supporting Information Tables S8 and S9, Figure 3, and Supporting Information Figure S5.

**Table 2.** Reliability of dFC parameters in terms of ICC

		**16’27” OWN data**		**14’24” HCP data**		**8’14” OWN data**		**7’12” HCP data**	
**116 ROIs**									
		**Uncentered**	**Centered**	**Uncentered**	**Centered**	**Uncentered**	**Centered**	**Uncentered**	**Centered**
**MDT**	**S**	0.01	0.23	**0.20**	**0.10**	0.62	0.17	**0.10**	0.00
		[−0.10, 0.30]	[−0.40, 0.56]	**[0.09, 0.33]**	**[0.02, 0.19]**	[−0.50, 0.80]	[−0.12, 0.42]	**[0.00, 0.20]**	[−0.07, 0.08]
	**I**	0.08	0.26	**0.16**	0.06	−0.23	0.13	0.00	**0.13**
		[−0.32, 0.45]	[−0.13, 0.59]	**[0.06, 0.29]**	[−0.04, 0.15]	[−0.57, 0.25]	[−0.30, 0.53]	[−0.06, 0.08]	**[0.06, 0.23]**
**Prev**	**S**	**0.51**	0.35	**0.47**	0.02	0.38	0.22	**0.39**	0.06
		**[0.15, 0.75]**	[−0.23, 0.58]	**[0.40, 0.55]**	[−0.07, 0.09]	[−0.02, 0.71]	[−0.21, 0.51]	**[0.31, 0.47]**	[−0.03, 0.14]
**ITI**		−0.10	0.25	0.09	**0.10**	−0.04	0.27	0.03	0.06
		[−0.21, 0.19]	[−0.18, 0.63]	[−0.00, 0.19]	**[0.01, 0.19]**	[−0.81, 0.76]	[−0.18, 0.56]	[−0.04, 0.12]	[−0.03, 0.15]
**Var**	**S**	**0.45**	0.21	**0.31**	**0.19**	0.43	−0.03	**0.23**	**0.09**
		**[0.05, 0.57]**	[−0.18, 0.60]	**[0.13, 0.49]**	**[0.09, 0.29]**	[−0.14, 0.59]	[−0.50, 0.35]	**[0.12, 0.35]**	**[0.01, 0.17]**
	**I**	**0.30**	0.24	**0.26**	**0.20**	−0.11	−0.19	**0.22**	0.06
		**[0.10, 0.45]**	[−0.07, 0.45]	**[0.16, 0.35]**	**[0.10, 0.28]**	[−0.29, 0.14]	[−0.60, 0.19]	**[0.12, 0.32]**	[−0.04, 0.15]
**442 ROIs**									
**MDT**	**S**	−0.37	−0.31	**0.27**	0.04	−0.01	0.31	**0.10**	−0.01
		[−0.62, −0.05]	[−0.52, 0.07]	**[0.12, 0.40]**	[−0.04, 0.13]	[−0.58, 0.14]	[−0.09, 0.54]	**[0.00, 0.19]**	[−0.09, 0.07]
	**I**	0.12	0.13	**0.25**	**0.12**	−0.03	0.44	**0.09**	0.07
		[−0.58, 0.19]	[−0.09, 0.36]	**[0.11, 0.39]**	**[0.03, 0.21]**	[−0.30, 0.29]	[−0.04, 0.60]	**[0.01, 0.19]**	[−0.02, 0.16]
**Prev**	**S**	**0.43**	0.30	**0.49**	0.04	0.27	0.01	**0.42**	0.06
		**[0.06, 0.71]**	[−0.06, 0.48]	**[0.41, 0.56]**	[−0.05, 0.12]	[−0.13, 0.60]	[−0.39, 0.32]	**[0.35, 0.49]**	[−0.02, 0.15]
**ITI**		0.02	−0.25	**0.18**	0.06	−0.05	0.35	0.02	−0.00
		[−0.59, 0.09]	[−0.45, 0.05]	**[0.06, 0.30]**	[−0.00, 0.14]	[−0.39, 0.28]	[−0.22, 0.64]	[−0.06, 0.11]	[−0.08, 0.10]
**Var**	**S**	0.01	0.26	**0.21**	**0.29**	0.13	0.17	**0.34**	**0.13**
		[−0.05, 0.55]	[−0.09, 0.49]	**[0.12, 0.31]**	**[0.20, 0.38]**	[−0.09, 0.39]	[−0.26, 0.44]	**[0.18, 0.49]**	**[0.04, 0.21]**
	**I**	0.31	0.22	**0.30**	**0.23**	**0.50**	0.19	**0.20**	**0.14**
		[0.04, 0.52]	[−0.13, 0.47]	**[0.18, 0.42]**	**[0.15, 0.32]**	**[0.03, 0.80]**	[−0.36, 0.50]	**[0.11, 0.29]**	**[0.05, 0.22]**

CI: 95% confidence interval based on bootstrapping (1,000 times); MDT: mean dwell time; Prev: prevalence; ITI: intertransition interval; Var: variability; S: state S; I: state I. **Bold:** confidence interval not including zero.

#### OWN data.

**Figure 4.**
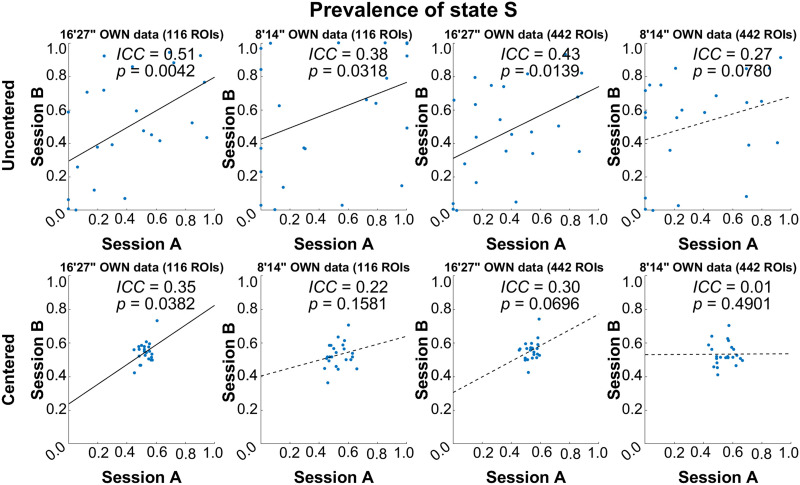
Scatterplots of prevalence between sessions for different pipelines to illustrate the reliability of OWN data. Solid line: *p* < 0.05; dotted line: *p* > 0.05.

The highest ICC value of 0.62 was obtained for *MDT*_*s*_ for the 8’14” OWN data; however, it appears to be driven by an outlier as the Spearman’s correlation was far from significant and the confidence interval was very wide (Table 2, Supporting Information Table S7, and Supporting Information Figure S6). Note that outliers did occur easily in uncentered data because dFC can be dominated by one or very few states only. The next highest ICC value of 0.51 was obtained for Prev for the 16’27” OWN data with the coarse atlas and without centering (Figure 4, Table 2, and Supporting Information Table S7). It is the only parameter that showed consistent reliability between pipelines (0.27 ≤ ICC ≤ 0.51 for uncentered data). Mean Prev values are plotted in Figure 3 as a bar graph with standard errors.

#### HCP data.

**Figure 5.**
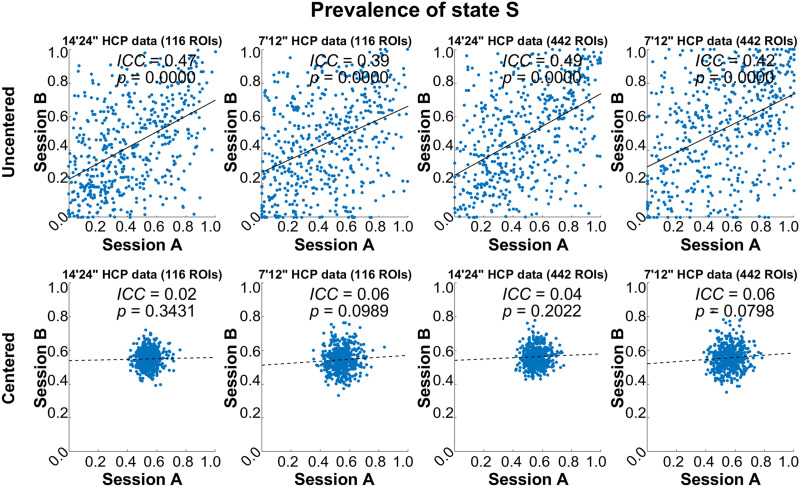
Scatterplots of prevalence between sessions for different pipelines to illustrate reliability of HCP data. Solid line: *p* < 0.05; dotted line: *p* > 0.05.

Reliabilities for HCP data are given in Table 2, Supporting Information Table S7, Figure 5, and Supporting Information Figure S7 and largely confirm the findings in OWN data. Prev_S_ for 14’24” HCP data based on the 116-ROI atlas showed an ICC of 0.47. The highest ICC value was 0.49 that was reached also for *Prev*_*S*_ by the pipeline with the same settings but with the finer atlas (Table 2). Moreover, Figure 3 shows that mean values of Prev_S_, particularly in HCP data, were higher and closer to 0.5 for centered data, the finer atlas, and shorter scan times.

### Correlations Between dFC Parameters

Spearman’s correlations between different dFC parameters within the same pipeline across subjects are given in Supporting Information Tables S10 and S11 for the coarse atlas and in Supporting Information Tables S12 and S13 for the fine atlas.

#### OWN data.

With some consistency over all pipelines (but not always significant), MDT_S_ and MDT_I_ were negatively correlated for the uncentered data, but positively correlated for the centered data. The Prev of one state was positively correlated with MDT of the same state and frequently negatively correlated with MDT of the other state for the uncentered data. For example, Prev_S_ was positively correlated with MDT_S_ with Spearman’s *rho* = 0.77 and negatively correlated with MDT_I_ *rho* = −0.76 for the long OWN data. This was consistent with the negative correlation between MDT_S_ and MDT_I_. For the centered data, the positive correlation of Prev with MDT of the same state was overall reduced and with MDT of the other state mostly not significant. This is consistent with the positive correlation between MDT_S_ and MDT_I_ for centered data. As to be expected, ITI was positively associated with MDT of both states, interestingly with stronger effects in the centered data. Var was positively correlated with MDT and Prev of the same state and negatively correlated with MDT and Prev of the other state for the uncentered data and much weaker, often not significant effects in the centered data.

#### HCP data.

The parameters from HCP data displayed similar characteristics as in OWN data.

### Comparing FC Distributions in Both Datasets

To identify differences in FC between the two datasets, distributions of static FC (i.e., mean dFC) values of session A for both atlases and datasets are shown in Supporting Information Figure S8 for the long scans with means and standard deviations. Both values are lower for HCP data than for OWN data for both atlases.

### Effects of GSR

To investigate whether clustering, when applying GSR, would still lead to one state being more segregated and another state being more integrated, we conducted graph-theoretical analyses for state centroid matrices based on 116 ROIs in the full-length, uncentered, GSR-corrected HCP data. While the resulting state centroids (see Supporting Information Figure S9) look visually much more similar to each other than without GSR, the graph-theoretical analysis (see Supporting Information Figure S10) shows that a more integrated and a more segregated state can still be clearly distinguished on the basis of their modularity and global efficiency (Supporting Information Figures S9 and S10). Comparing the state centroids quantitatively in terms of ICC and CD also shows that all centroids are much more similar and that reliability after removing the mean of the state centroids is much lower when compared with no GSR: ICC = 0.94/0.95 versus 0.75/0.89 for matching S/I states, respectively (see Supporting Information Table S14 and Table 1C). The reliability of the dFC parameters with GSR (see Supporting Information Table S15) was very similar to the values without GSR (Table 2). Notably, we also evaluated the reproducibility^1^ of the parameters across pipelines, that is, with and without GSR (Supporting Information Table S16), and found similar values as for the test-retest reliability (Table 2), for example, ICC = 0.53/0.56 for Prev in sessions A/B, respectively (see Supporting Information Table S16).

### Comparison With a Five-State Model

Both the two-state and five-state models showed high similarity of centroids for back-projecting A1 ➔ B1 (*ICC* ≥ 0.92 when removing the mean of the state centroids; Supporting Information Table S17). The corresponding state centroids are visually hard to distinguish (Supporting Information Figures S13 (1) and S14 (1)). However, when reclustering A1 versus B1, at least some of the clusters for *k* = 5 look visibly different (Supporting Information Figure S14 (2)), which is not the case for the two-state model (Supporting Information Figure S13 (2)). Quantitatively, ICC ranges from 0.22 to 0.87 for *k* = 5 while values are ≥0.88 for the two-state model (Supporting Information Figure S15, Supporting Information Tables S17 and Table S18 for CD). Of note, the state matching under A1 versus B1 condition displayed a conflict for States 1 and 5, that is, both States 1 and 5 in A1 showed the highest similarity with State 1 in B1. The reliability of the dFC parameters was basically unaffected by the two different clustering approaches for the two-state model (Supporting Information Table S19), but Prev of State 5 dropped significantly for the five-state model with reclustering. The other Prev values also dropped but not significantly (Supporting Information Table S20). The power of the analysis was not sufficient to detect further significant differences.

## DISCUSSION

For the proposed two-state model, our graph-based analysis confirmed that clustering results in an integrated brain state with low modularity and high global efficiency and a segregated brain state with opposite properties. Our analysis, using eight different pipelines, in HCP data confirmed our previous findings in the OWN data. See the Supporting Information Discussion section for a comparison of the two datasets and the correlation between different dFC parameters.

### Reliability of Cluster Centroids

Consistent with Choe and colleagues’ (2017) study, we found high reliability of the paired clusters between sessions for both states based on all analysis strategies. In fact, for uncentered data, the ICC was > 0.99 for HCP data even for the short scan length compared with values between 0.93 and 0.99 for OWN data, which is likely a consequence of the much larger number of subjects, that is, state instances, included in the clustering/back-projection process for HCP data. This indicates that reliability of the centroid estimation is not yet at ceiling with only 16’27” × 23 subjects of data. This effect is even noticeable in HCP data when removing the mean matrix of state mean, which reduces the ICC from 0.99 to 0.94–0.97 for the longer HCP scan and almost the same effects (0.96–0.98) on the shorter HCP scan. Note that ICC values for uncentered data overestimate reliability in the sense that ICC values are even high between the two different states (ICC ≤ 0.79), which indicates that the two states differ primarily in strength, not the direction of the correlations. Supporting Information Figure S3 shows the effect of centering on the distances to each centroid. Centering increased the dissimilarity between the unpaired states. It also shifted the Prev of either state toward 0.5 (see Figures 4 and 5) and, thus, reduced the number of subjects that remained in the same state for the whole scan, that is, never change states.

Values for the centered data are comparable with those after removing the session mean for the uncentered data. A finer atlas reduced ICC values in most cases (Table 1). These effects can also be observed when looking at the similarity of the clusters in terms of CD (Supporting Information Table S5). As expected, removing the session mean from the uncentered data increased CDs of the unpaired clusters, because the connectivity vectors are moved toward the center of the clustering space with clusters largely falling into opposite quadrants.

The structure of the two states based on the uncentered data is visually consistent with other dynamic two-state patterns in the whole brain at rest for not only healthy people (Choe et al., 2017) with eyes opened/closed (Weng et al., 2020) and different ages (X. Yang et al., 2023) but also patients with Parkinson’s disease (Fiorenzato et al., 2019; Kim et al., 2017), major depressive disorder (Zheng et al., 2022), and dementia with Lewy bodies (Ma et al., 2019). This consistency in state structure between studies is encouraging because, if the structure had varied, statements about brain organization would lack a common ground for comparison. However, this comparability may be somewhat reduced for short scan lengths, few subjects, and fine atlases.

### Reliability of dFC Parameters

Prev showed the highest reliability across analyses (i.e., 6/18 pipelines; see Table 2 and Supporting Information Table S7), which is consistent with existing findings (Abrol et al., 2017; Choe et al., 2017; Smith et al., 2018). This is especially true for all four HCP pipelines without centering, which provide the highest signal-to-noise ratio (ICC confidence interval). This is also in line with the theoretical consideration that it is computed based on the complete scan time and does not necessarily depend on the number of state switches, that is, it benefits from averaging over all instances of a particular state. MDT is, on the other hand, derived in average from only half the scan time with the number of state transitions being an additional source of variance (an argument that is extendible to *k* > 2). Thus, in the absence of a deeper theoretical framework of how brain integration is reflected differentially in Prev, MDT, and ITI, the parameter Prev is recommended for the formulation of hypotheses related to correlations between dFC measures of functional brain integration/segregation and other subject characteristics as higher reliability provides higher power to detect existing associations.

Reliability of the dFC parameters is, at best however, intermediate with an ICC = 0.49 for Prev for uncentered, 14’24” HCP data, and the fine atlas. Shorter scans and centering reduced this value. Centering in particular, while conceptually nice to remove between-subject differences in static connectivity, strongly reduced reliability. As the ICC measures the fraction of the between-subject variance of the total variance, this can be explained by a substantial reduction in between-subject variance when removing the differences in static connectivity without effecting the within-subject variance very much. Importantly, while shorter scan lengths may be thought of as enhancers of temporal noise introducing only minimal shifts in dFC parameter rank, centering needs to be recognized as an alteration of the brain state concept that not only introduces a potentially drastic shift of, for example, Prev toward 0.5 but also will reorder ranks substantially. In a way, it almost randomizes the dFC parameters given that reliabilities drop drastically (see Table 2). The reliability of either MDTs was less than of Prev with the largest significant ICC = 0.27 for MDT_S_ and 0.25 for MDT_I_ both for the uncentered, 14’24” HCP data with the fine atlas. Generally lower ICC values for MDT compared with Prev were expected for the above reason.

Several reports suggested higher reliabilities (ICC = 0.56 [Choe et al., 2017] and ICC ≤ 0.47 [Smith et al., 2018]) of the MDTs than observed in this study. This may be due to a sensitivity of MDT to methodological differences (e.g., direct clustering vs. two-level clustering and ROI selection based on ICA [independent component analysis] vs. atlas-based) (Abrol et al., 2017; Choe et al., 2017; Smith et al., 2018). In addition to the MRI sequence parameters, both the number of regions and the ICA-based noise filtering may also have an impact here. The reliabilities of the ITI parameter were low and not significant for many pipelines. Significant ICC values for ITI = 0.09–0.18 were only reached for the longer HCP data. Reliabilities for the Var parameter were ICC = 0.21–0.31 for the longer, uncentered HCP data and not as strongly reduced by centering as for other parameters.

Several considerations are relevant with respect to centering. To obtain the highest reliability for Prev, centering is not recommended. It will eliminate a large portion of the between-subject variance, which is a disadvantage for cross-sectional analyses. It will also effect the interpretation of the brain state and may make the labels “integrated” versus “segregated” less meaningful, that is, a subject that displays more integration (high connectivity) over all frames may lose this label when such between-subject differences in overall (static) connectivity are removed. Not to center, however, also has considerable disadvantages: (a) Without centering, static and dynamic effects are mixed, that is, differences in static connectivity may drive between-subject differences rather than the dynamics, and (b) centering could reduce noise if differences in static connectivity are fluctuating on the time scale of hours to days without affecting the dynamics. Thus, centering may be of interest for longitudinal studies over longer periods.

### Effects of GSR

GSR is a controversial preprocessing step in rs-fMRI (Murphy & Fox, 2017). We tend not to use it, following the arguments presented in Gotts et al. (2013), especially because it is known that it can induce false negative correlations. However, given that many labs use it, presumably because it improves correlations with behavior (Li et al., 2019), we investigated its effect on the two-state model. As expected, both matrices show much more negative correlations (see the right part in Supporting Information Figure S9). The two states look visually much more similar than without GSR, which is also reflected in a stronger overlap between centroids in the graph measures modularity and global efficiency when bootstrapping over subjects (see Supporting Information Figure S10). This also leads to lower state reliability (see Table 1 vs. Supporting Information Table S14). Nevertheless, it is still possible to identify a more integrated versus a more segregated state in Supporting Information Figure S14. The reliability of the dFC parameters is not affected (see Supporting Information Tables S17 and S18). However, the reproducibility of the dFC parameters with versus without GSR is only about ICC = 0.50 (see Supporting Information Table S15) at best (for Prev). This means that the two Prev measures share only about 25% in variance and that the effect of applying GSR on the dFC parameters is similar to a retest of the same subjects. We conclude that GSR does not change the reliability of the dFC parameters but that it could and likely will affect correlations with behavior.

### Limitations and Future Works

There are some limitations of the current work that should be addressed in the future: (a) Because of focusing on effects of Var in connectivity, we employed back-projection in the second session instead of reclustering, which avoids introducing additional effects due to cluster-centroid differences between groups, which complicates an interpretation and is more severe for larger *k* (see Supporting Information Figure S15). (b) The two-state model may not be optimal to maximize reliability of a specific state or identify group differences in such specific states. Thus, models with more states and other dynamic methods, for example, coactivation analyses (Karahanoğlu & Van De Ville, 2015), could show superiority in detecting specific differences in particular networks or conditions. However, in the absence of concrete hypotheses about what states could be affected, we believe that two states are a good choice for the reasons given in the introduction. We also demonstrated the advantages of the two-state model over a five-state model when no theoretical considerations favor more states, namely, the reduced reliability of the cluster centroids and related dynamic parameters when reclustering (see Supporting Information Figures S15 and S16, Supporting Information Tables S17, S18, S19, and S20). We are not claiming in this context that two brain states are a realistic description of the number of states available to the brain as this number is obviously vast, which is reflected in numerous task-based activation patterns and FC patterns. In contrast, we believe that the choice of the number of states is largely a choice of the investigator made to understand higher-order principles and that the term “state” is to be seen as a category that dFC matrices are sorted into, not as a physiological reality associated with some true number of possible brain states. (c) Our clustering approach does not allow for mixed states that permit a subject to be simultaneously in multiple overlapping states (Leonardi et al., 2014; Miller et al., 2016). (d) Many more processing pipelines that may improve reliability are imaginable, for example, more atlases with different granularities, different noise filters, different MR sequences, coactivation versus temporal correlation, and so forth. While we did not find that study population, preprocessing (minimal in OWN data or extensive in HCP data), GSR, or the choice of the atlas had a major impact on the reliability of the data, it should be noted that the differences in state assignment can still be substantial, which is demonstrated by the only moderate correlation within the same parameter with and without GSR correction. This means that pipeline selection may still impact potentially observable correlations with behavior or personality.

### Conclusions

In summary, we found high reliability of clustering results with a two-state model but only medium to low reliability of the dFC parameters. We recommend state Prev as the most reliable parameter to formulate hypotheses related to functional integration and segregation in the whole brain. Additionally, because shorter scan lengths and centering decrease reliability, such effects should be considered when conducting dFC studies.

## ACKNOWLEDGMENTS

We thank all subjects for their study participation The authors gratefully acknowledge the computing time provided to them on the high-performance computers Taurus and Barnard at the Zentrum für Nationales Hochleistungsrechnen at the Technische Universität Dresden (NHR@TUD) (∼100,000 CPUh). This is funded by the Federal Ministry of Education and Research and the state governments participating on the basis of the resolutions of the GWK for the national high-performance computing at universities (www.nhr-verein.de/unsere-partner). We are also thank the DFG-supported Open Access Publication Fund by the Technische Universität Dresden managed by the Sächsische Landesbibliothek –Staats- und Universitätsbibliothek Dresden.

## SUPPORTING INFORMATION

Supporting information for this article is available at https://doi.org/10.1162/netn_a_00437.

## AUTHOR CONTRIBUTIONS

Xiaojing Fang: Data curation; Formal analysis; Investigation; Software; Validation; Visualization; Writing – original draft; Writing – review & editing. Michael Marxen: Data curation; Funding acquisition; Methodology; Project administration; Resources; Supervision; Validation; Writing – review & editing.

## FUNDING INFORMATION

Michael Marxen and Xiaojing Fang, Deutsche Forschungsgemeinschaft (https://dx.doi.org/10.13039/501100001659), Award ID: 178833530. Michael Marxen and Xiaojing Fang, Deutsche Forschungsgemeinschaft (https://dx.doi.org/10.13039/501100001659), Award ID: 402170461.

## DATA AVAILABILITY STATEMENT

Code and signal time courses to reproduce our results are available on https://osf.io/mu3st. The HCP data are publicly available at HCP Young Adult - Connectome (humanconnectome.org).

## ETHICS APPROVAL AND PATIENT CONSENT STATEMENT

The study was approved by the Ethics Review Board of the Technische Universität Dresden (EK45022016). All subjects signed informed consent forms after receiving a detailed description of the experiment.

## Note

^1^ Throughout the manuscript, we use the term “reliability” to mean “test-retest reliability.” We use “reproducibility” here, because we are evaluating “reproducibility across pipelines” in this case. Both concepts may be quantified by the same correlation measures.

## Supplementary Material



## TECHNICAL TERMS

Functional connectivity (FC): A concept that describes the correlation or synchronicity of fMRI signals between different brain regions.
Dynamic functional connectivity (dFC): A conception presuming that FC changes on the time scale of seconds, rather than being static for minutes.
Resting state: Condition of quiet repose, wakefulness, and relaxation with eyes closed or open, without specific tasks or cognitive engagement.
Test-retest reliability: Consistency of results redrived from repeated measurements within the same subject or group.
Functional magnetic resonance imaging (fMRI): Noninvasive neuroimaging technique that infers neural activity by detecting variations in blood oxygenation in the brain.
Functional integration: Coordinated interactions among specialized brain regions/networks that integrate information, enabling coherent outputs and unified brain function.
Functional segregation: The brain’s ability to divide tasks and processes among specialized regions or networks.
Graph analysis: Mathematical analysis employing graph theory to represent a brain system as a network composed of nodes and interconnecting edges.
Spontaneous brain activity: Brain’s intrinsic neural activity that occurs in the absence of any external stimuli or intentional cognitive tasks, for example, at rest.
Functional brain atlas: Detailed parcellation or map of the brain based on regional function and/or functional connectivity.
