# Supplementary material for: Test-retest reliability of dynamic functional connectivity parameters for a two-state model
Source: Netw Neurosci. 2025 Mar 20;9(1):371–91. doi: 10.1162/netn_a_00437 (PMC11949578; doi:10.1162/netn_a_00437)
Supplement: Supplementary file 1 [file netn-9-1-371-s001.pdf]

Supplemental Information

for

Test-retest reliability of dynamic functional connectivity parameters for a two-state model

Xiaojing Fang and Michael Marxen*

Department of Psychiatry, Technische Universität Dresden, Dresden, Germany

*** Correspondence:** Michael Marxen, Section of Systems Neuroscience, Technische Universität Dresden, Würzburger Straße 35, 01187 Dresden, Germany, Email: michael.marxen@tu-dresden.de

Contains Supplemental Methods, Supplemental Results (Figures S1-S16 and Tables S1-S20) and Supplemental Discussion.

1. Supplementary Methods (sections as in main manuscript)

1.1. Participants, data acquisition, and preprocessing

1.1.1. OWN data

We collected this data at the Neuroimaging Center of the Technische Universität Dresden. Twenty-five healthy young adults were recruited from a volunteer database. Inclusion criteria were no magnetic-resonance contraindications, normal or correctable-to-normal vision and an age between 20 and 35 years. For the analysis, one participant was excluded because of incomplete MRI data, another because of severe, prefrontal signal dropout despite field map correction. Therefore, we analyzed the data of 23 subjects [mean ± standard deviation (SD) of age: 24.0 ± 3.8 years, 16 females]. Participants received financial compensation of €40 for full participation in two sessions. The study was approved by the Ethics Committee of the Technische Universtiät Dresden (EK 4012016) and all participants signed informed consent forms after receiving a detailed description of the experiment. To evaluate reliability, a repeated-measure (i.e., test - retest) design was used. Study participants were scanned twice with the same scanning protocol with approximately one week between scanning sessions. To eliminate potential session order effects, we generated two sets of data (i.e., session A and B) by randomly assigning the first acquisition session from 12 participants and the second acquisition session from the remaining 11 participants to session A, while the respectively paired scans were assigned to session B.

Imaging data were collected on a Siemens 3 T Magnetom Trio Tim scanner (Siemens, Erlangen, Germany) equipped with a 32-channel head coil. In each session, structural T1-weighted data were acquired after brief localizer scans (~1 min), using a 3D magnetization-prepared rapid gradient echo (MPRAGE) sequence with the parameters of repetition time (TR) 2400 ms, echo time (TE) 2.19 ms, voxel size 0.85 mm × 0.85 mm × 0.85 mm, inversion time 1000 ms, field of view (FOV) 272 mm × 272 mm, flip angle 8°, matrix 320 × 320, slice thickness 0.85 mm, band width (BW) 210 Hz/Px and 240 slices. T2-weighted data were acquired in each session with the parameters of TR 3200 ms, TE 565 ms, voxel size 0.85 mm × 0.85 mm × 0.85 mm, FOV 272 mm × 272 mm, flip

angle 120°, matrix 320 × 320, thickness 0.85 mm, BW 744 Hz/Px and 240 slices. We acquired a B0 field map with parameters of TR 832 ms, TE-1 5.19 ms, TE-2 7.65 ms, voxel size 2.0 mm × 2.0 mm × 2.0 mm, gap 0 mm, FOV 208 mm × 208 mm, flip angle 58°, matrix 104 × 104, BW 278 Hz/Px, and 78 slices. Then, we collected rs-fMRI data based on a multi-band axial (T > C ~ -17°) 2D EPI sequence (Moeller et al., 2010) with the parameters of TR 987 ms, TE 32.6 ms, voxel size 2.0 mm × 2.0 mm × 2.0 mm, slice gap 0 mm, FOV 192 mm ×192 mm, flip angle 62°, matrix 96 × 96, BW 1860 Hz/Px, 72 interleaved slices, and 1000 volumes. The two rs-fMRI scans lasted approximately 16' 27'' each (mean ± SD of the interval between the two scans: 8.87 ± 4.84 days). Additionally, we acquired diffusion-weighted images not relevant to this study. All participants received foam padding for head-movement reduction and earplugs for hearing protection and were instructed to close their eyes and to try not to fall asleep.

1.1.2 HCP data

The other dataset used for this study is from the HCP S1200 release (Van Essen et al., 2013) whose data were acquired on Siemens connectome-Skyra 3T scanner with a 32-channel head coil. In this dataset, participants completed two scanning sessions on two separate days. After brief localizer scans, T1w (MPRAGE) structural data were acquired during each session with TR 2400 ms, TE 2.14 ms, voxel size 0.7 mm× 0.7 mm × 0.7 mm, inversion time 1000ms, FOV 224 mm × 224 mm, flip angle 8°, matrix 320× 320, slice thickness 0.7 mm, band width (BW) 210 Hz/Px and 256 slices. For the rs-fMRI data (acquisition time: 14' 24'') in each session, a simultaneous multi-slice pulse sequence with an acceleration factor of eight (Ugurbil et al., 2013) was used with TR 720 ms, TE 33.1 ms, voxel size 2.0 mm × 2.0 mm × 2.0 mm, FOV 208 mm ×208 mm, flip angle 52°, matrix 96 × 96, BW 2290 Hz/Px, 72 interleaved slices, and 1200 volumes. Participants were instructed to keep their eyes open and fixated on a crosshair on the screen. In each session, phase encoding directions for the two runs were alternated between right-to-left and left-to-right directions. We used the data with the left-to-right direction and limited our analyses to 501 participants (mean ± SD of age: ~28.9 ± 3.6 years, 265 females) who completed the full rs-fMRI protocol in the dataset.

1.1.3 Preprocessing

OWN data

The following preprocessing pipeline using fMRIPrep 1.2.5 (zenodo.org/record/4252786#.X7TzMGhKhPZ) based on Nipype 1.1.6 (zenodo.org/record/4035081#.X7Ty32hKhPY) (Esteban et al., 2019) was employed: skull stripping, field map correction, co-registration of the structural and functional images, head-motion estimation and correction, spatial normalization to MNI152NLin2009cAsym space. All subjects met our inclusion criteria of less than 7.5% of frames with framewise displacement (Power, Barnes, Snyder, Schlaggar, & Petersen, 2012) ≥ 0.5 mm. Then, the nuisance covariates (i.e., six head motion parameters, mean signals of cerebral spinal fluid and white matter using conservative masks) were regressed out from the preprocessed data. The effects of low-frequency drift and high-frequency physiological noise were reduced by using a band-pass filtering [1/(w) to 0.1 Hz] in time (Leonardi & Van De Ville, 2015). Here, we specified w (i.e., the window width in TR for the sliding-window analysis) as 40 TR (Zhang, Baum, Adduru, Biswal, & Michael, 2018), which resulted in a lower frequency boundary of 0.025 Hz.

HCP data

We used the preprocessed rs-fMRI data with FIX cleaning (Glasser et al., 2013; Smith et al., 2013) from the HCP1200 data set and regressed out the six head motion parameters as well as mean cerebral spinal fluid and white matter signals as for the OWN data. The band-pass filtering was employed with the consistent high-frequency boundary of 0.1 Hz and lower frequency boundary of around 0.025 Hz as used in OWN data, which corresponds to a window width of 55 TR.

Employed brain atlases

The first version of Automated Anatomical Labeling atlas (AAL) (Tzourio-Mazoyer et al., 2002) and a joint atlas (Schaefer et al., 2018; Shine et al., 2016) were employed in this study to explore the reliability of dFC parameters on different spatial scales. The former consists of 116 ROIs and was grouped into nine networks/regions based on

Yeo's seven functional networks of the cerebral cortex (Yeo et al., 2011) – visual, sensory-motor, dorsal attention, ventral attention, limbic, fronto-parietal, and default mode network - plus the basal ganglia network, and the cerebellar network. *AAL ROIs were matched to Yeo's networks based on the maximum of a parcel's overlap with each network*

(github.com/ThomasYeoLab/CBIG/blob/master/stable_projects/brain_parcellation/Yeo2011_fcMRI_clustering/1000subjects_reference/Yeo_JNeurophysiol11_SplitLabels/MNI152/)."

The joint atlas consisted of 442 ROIs: 400 cortical parcels of the Schaefer atlas (Schaefer et al., 2018), 14 subcortical regions from the Harvard-Oxford subcortical atlas, i.e. the left and right thalamus, caudate, putamen, ventral striatum, globus pallidus, amygdala, and hippocampus, and 28 cerebellar regions from the spatially unbiased infra-tentorial template atlas (Diedrichsen, Balsters, Flavell, Cussans, & Ramnani, 2009). This joint atlas was grouped into the same nine networks (see Supplementary Tables S1-S3).

2. Supplementary Results

We chose k = 2 clusters out of theoretical considerations. Nevertheless, we evaluated whether our choice was optimal in terms of the silhouette statistic for OWN data in session A (Fig. S1). Although the silhouette statistics that resulted from k = 2 to 20 showed more fluctuations for the uncentered data than for the centered data in all the strategies, the results consistently showed an optimal cluster number of k = 2.

Fig S1. Silhouette statistics (based on session A) for different time-length, atlases and uncentered/centered OWN data.

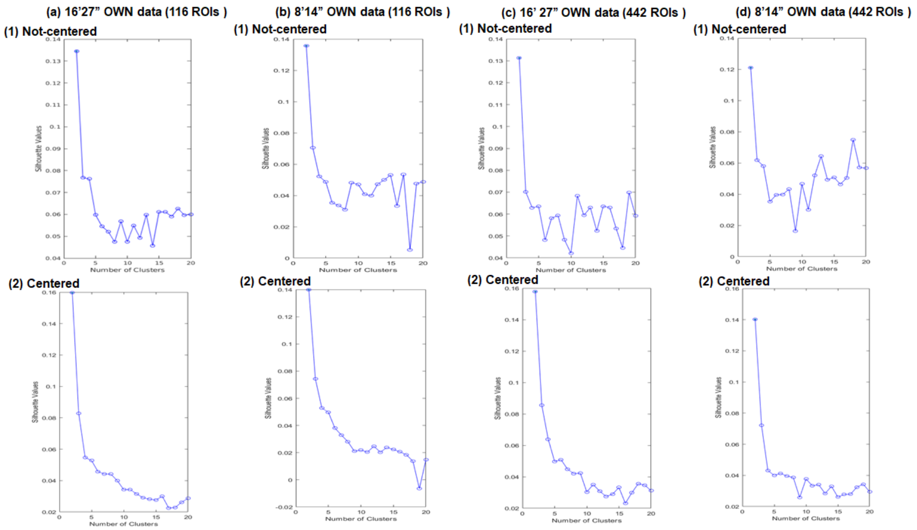

Fig S2. Cluster centroids (k = 2) of dFC matrices based on 442 ROIs for different time-length and uncentered/centered OWN data. For session A, k-means clustering was used for state assignment, while back projection results are shown for session B. Displayed are group means of the within-run means (N1: visual network; N2: sensory-motor network; N3: dorsal attention network; N4: ventral attention network; N5: limbic network; N6: fronto-parietal network; N7: default mode network; N8: basal ganglia network; N9: cerebellum network).

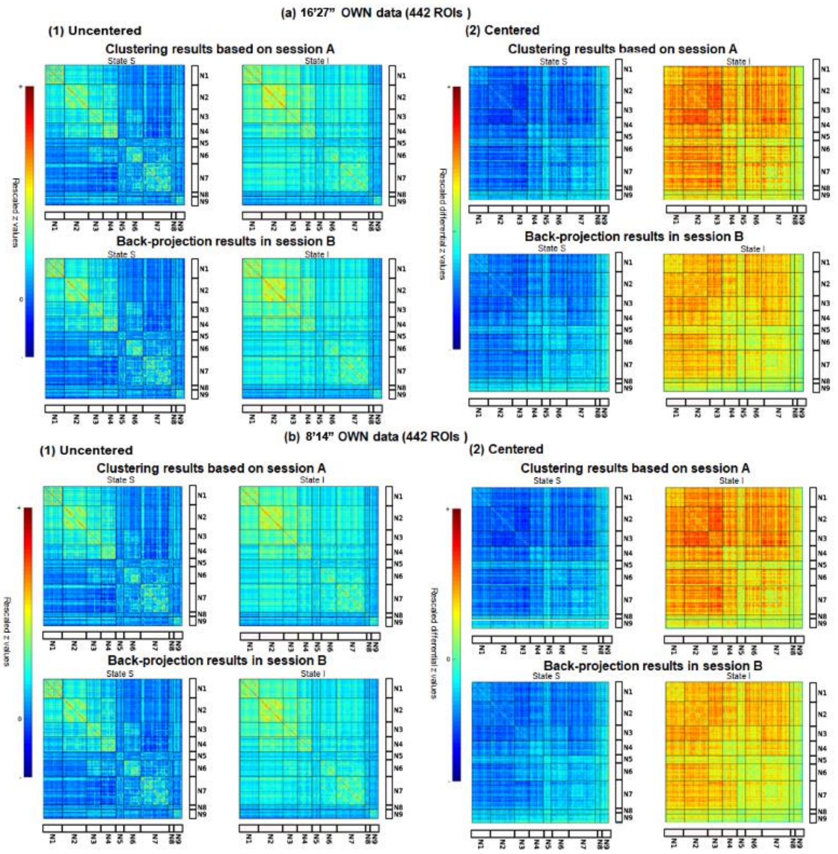

Fig S3. Plot of the cosine distances (by definition element of [0;2]) of state matrices to the centroid of states I on the x-axis and state S on the y-axis (OWN data, 16’27’’, 116 ROIs, session A). (state S – red; state I – blue) dFC example matrices (centered data) near the boundary between the clusters and far away from the boundary are also shown. Each dot represents a matrix computed from one time window of one of 23 subjects. The diagonal line of identity separates the two clusters. A cosine distance of 1 means that the angle between two-state vectors in the state space is 90°. Centering introduces a strong anti-correlation into the distances as the state space is now centered on zero, which means that the vectors that represent the centroids are pointing in opposite directions, i.e. have a cosine distance near 2. In consequence, the cosine distance of any matrix to state I is approximately [2 – distance to state S]. For the uncentered data, the angle between the centroid vectors is much less than 90°, i.e. a cosine distance < 1 because the data is far away from the coordinate center (zero), and all distances are consequently smaller than 1.

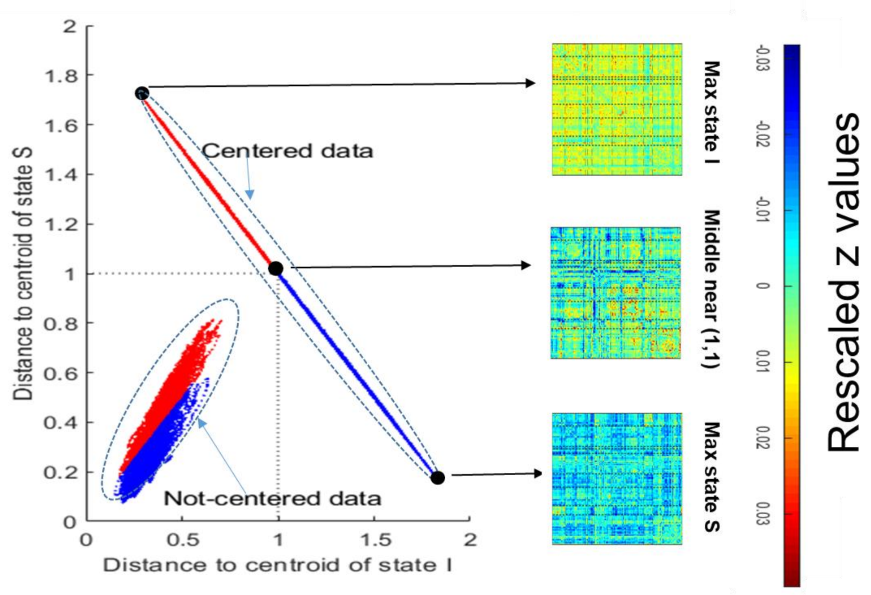

Fig S4. Cluster centroids (k = 2) of dFC matrices based on 442 ROIs for different scan length and uncentered/centered HCP data. For session A, k-means clustering was used for state assignment, while back projection results are shown for session B. Displayed are group means of the within-run means (N1: visual network; N2: sensory-motor network; N3: dorsal attention network; N4: ventral attention network; N5: limbic network; N6: fronto-parietal network; N7: default mode network; N8: basal ganglia network; N9: cerebellum network).

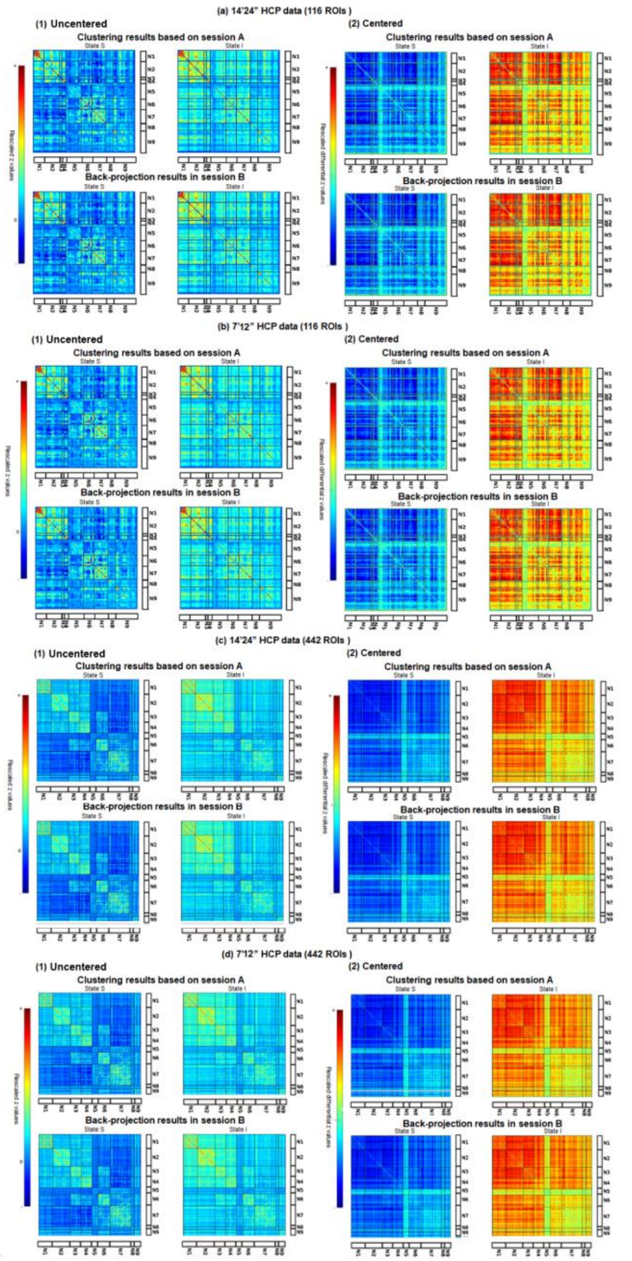
(a) 14'24" HCP data (116 ROIs)
(1) Uncentered
(2) Centered
Clustering results based on session A
Back-projection results in session B
State S
State I
(b) 7'12" HCP data (116 ROIs)
(c) 14'24" HCP data (442 ROIs)
(d) 7'12" HCP data (442 ROIs)

Fig S5. Bar plots of mean dwell time (*MDT*) and inter-transition time (*ITI*) in units of seconds, and state variability (*Var*) based on different pipelines with standard error of the mean.

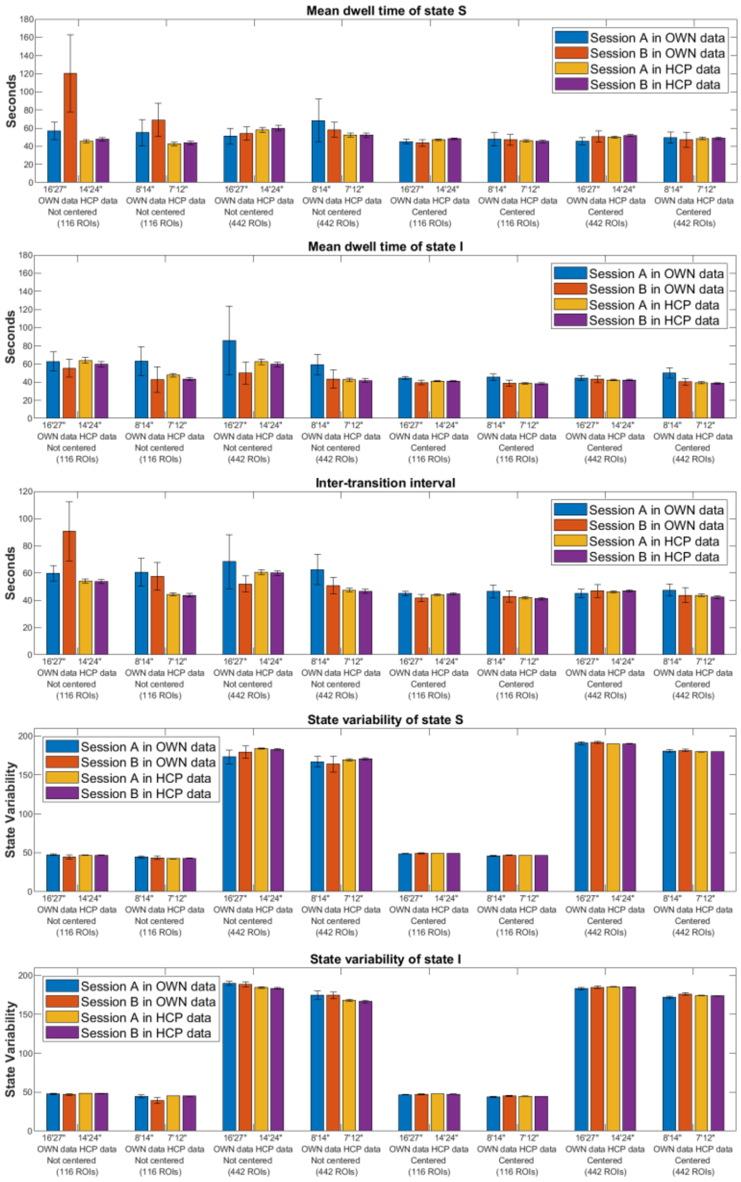

Fig S6. Scatter plots of mean dwell time (*MDT*) and inter-transition time (*ITI*) in units of seconds, and state variability (*Var*) based on different pipelines of OWN data with *ICC* and *p* values. Solid line: $p < 0.05$; dotted line: $p > 0.05$.

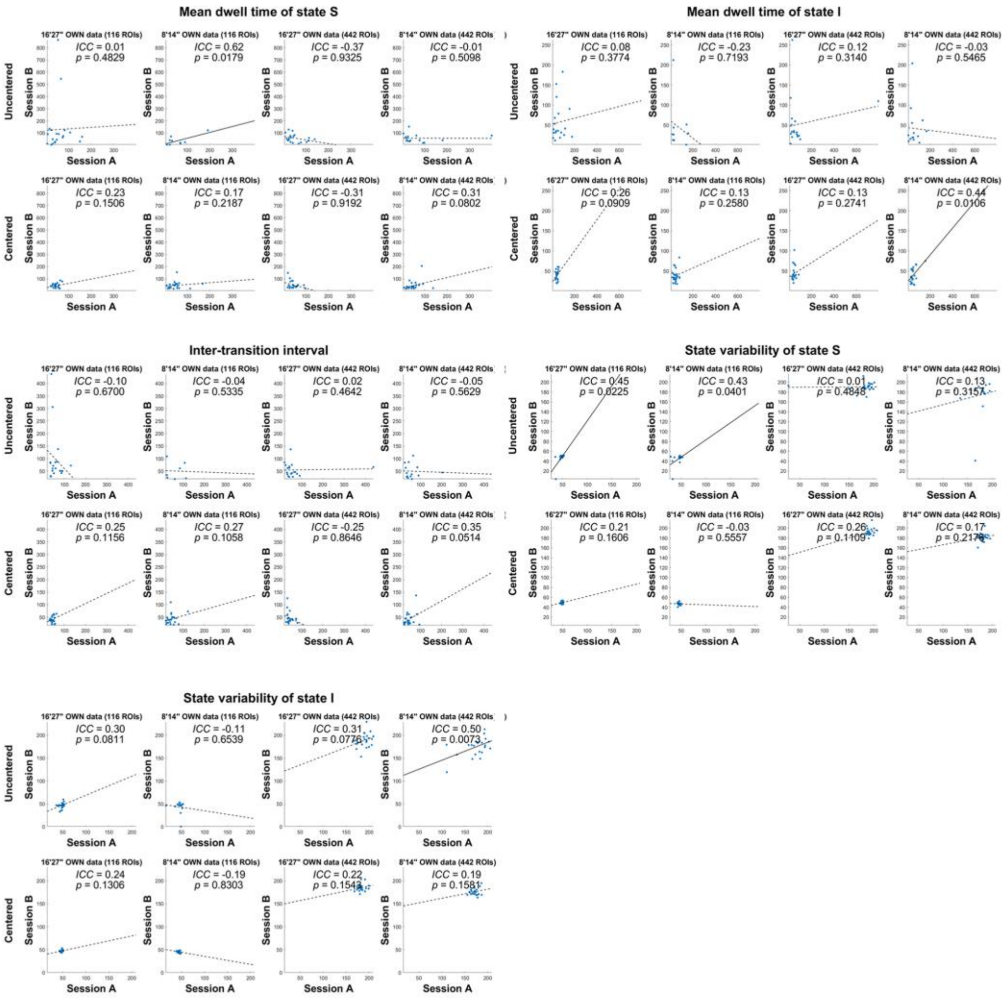

Fig S7. Scatter plots of mean dwell time (*MDT*) and inter-transition time (*ITI*) in units of seconds, and state variability (*Var*) based on different pipelines of HCP data with *ICC* and *p* values. Solid line: $p < 0.05$; dotted line: $p > 0.05$.

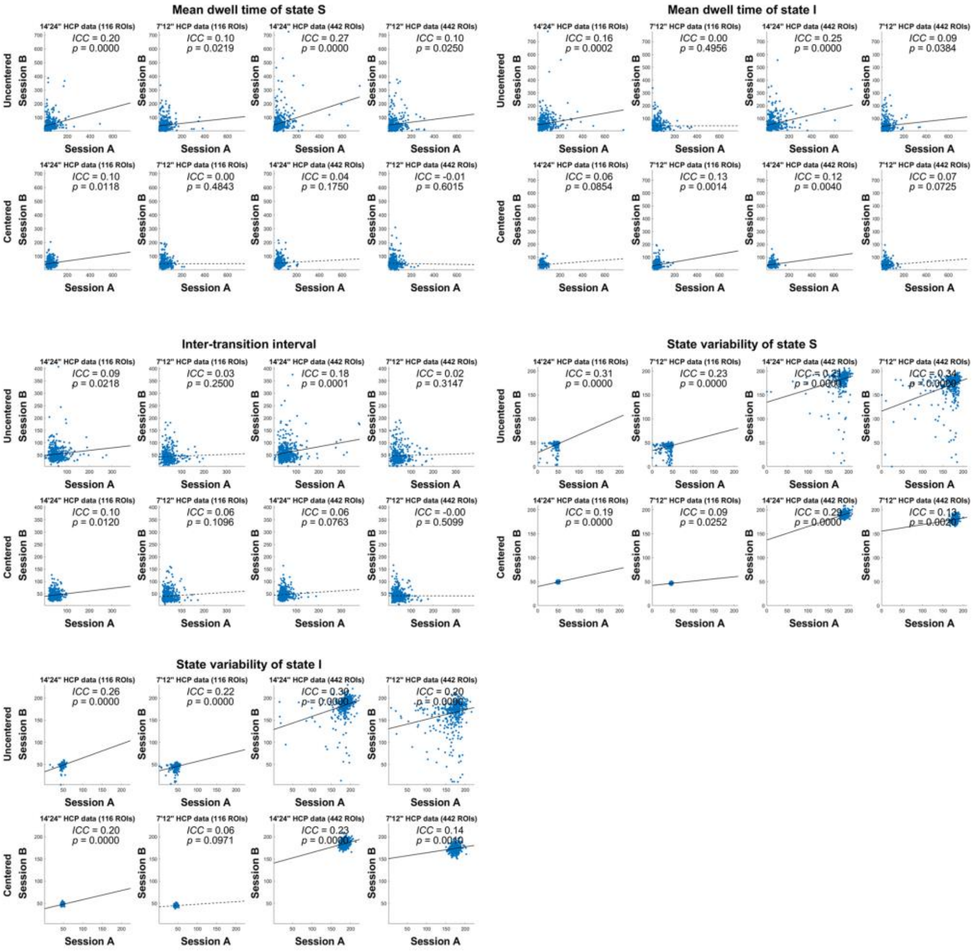

Fig S8. Summaries of static functional connectivity based on session A across atlases and datasets. (a) and (b) are the histograms for the distributions. SD: standard deviation. c) is the mean values of different pipelines (i.e., bars) with 95% confidence interval computed based on bootstrapping with 1000 random samples.

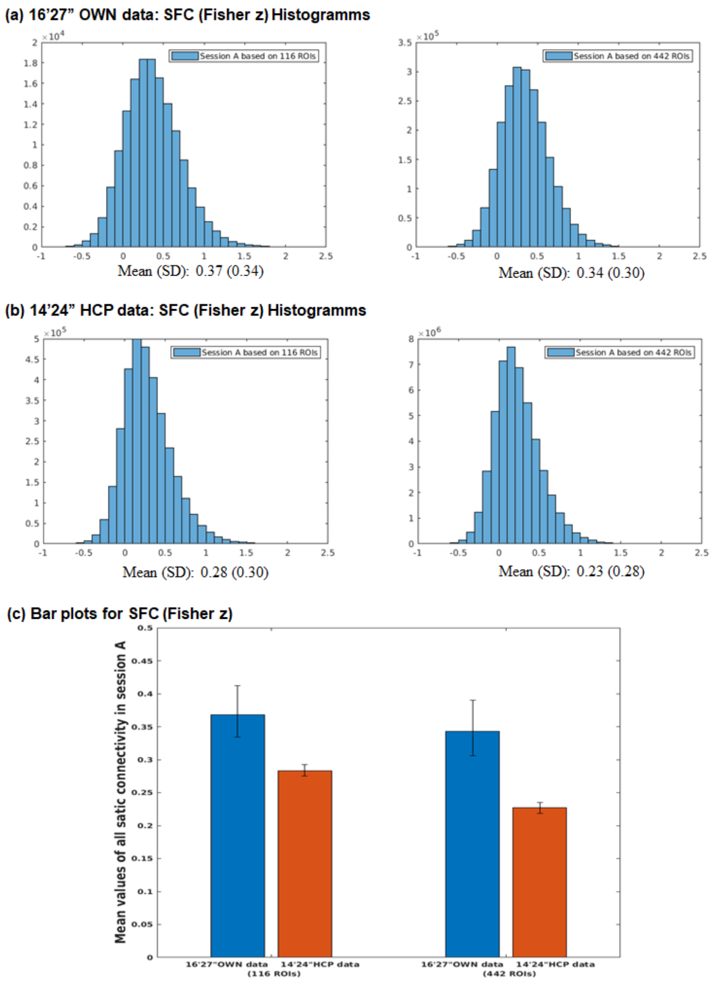

Fig S9. Cluster centroids (k = 2) of dFC matrices based on long HCP data and 116 ROIs with and without global signal regression (GSR).

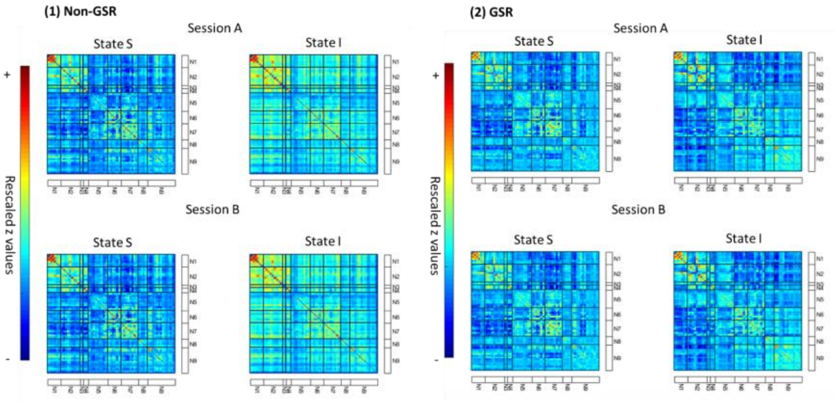

Fig S10. Global graph-theoretical measures of cluster centroids based on long HCP data and 116 ROIs when applying global signal regression. Btsp.: repeated clustering of 1000 bootstrapping samples from the subject pool; Ses.: session.

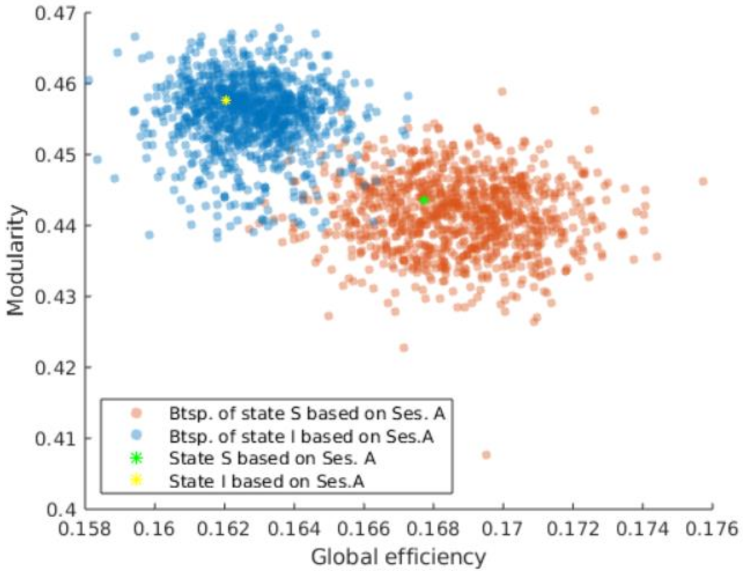

Fig S11. Scatter plots of dFC parameters between 116 ROIs with and without global signal regression (GSR) uncentered HCP data. Solid line: $p < 0.05$; dotted line: $p > 0.05$.

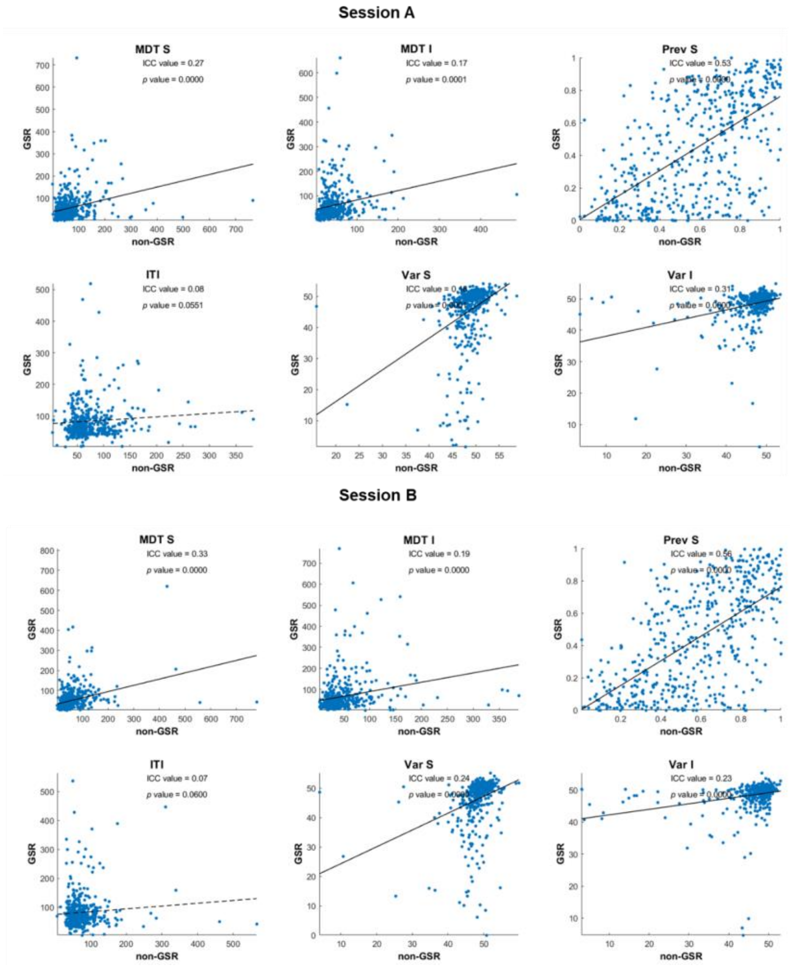

Fig S12. Scatter plots of dFC parameters between sessions in uncentered, 116 ROIs HCP data with global signal regression. Solid line: $p < 0.05$; dotted line: $p > 0.05$.

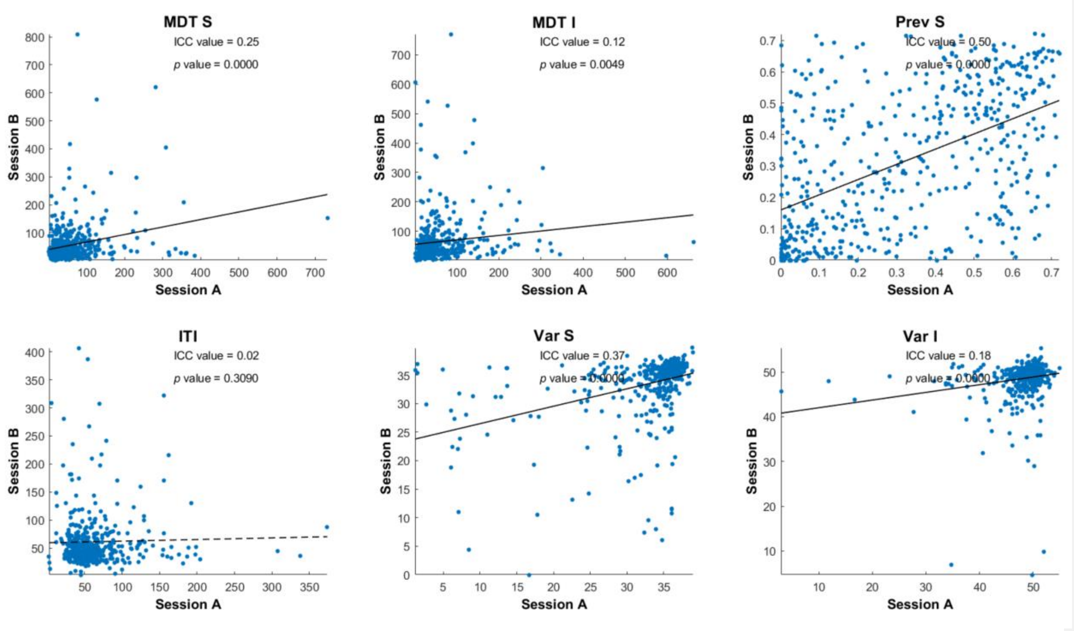

Fig S13. Cluster centroids (k = 2) of the dFC matrices based on 116 ROIs, long, uncentered HCP data. A1→B1: Clustering session A1 and back projection on session B1; A1 vs. B1: Clustering session A1 and B1 individually.

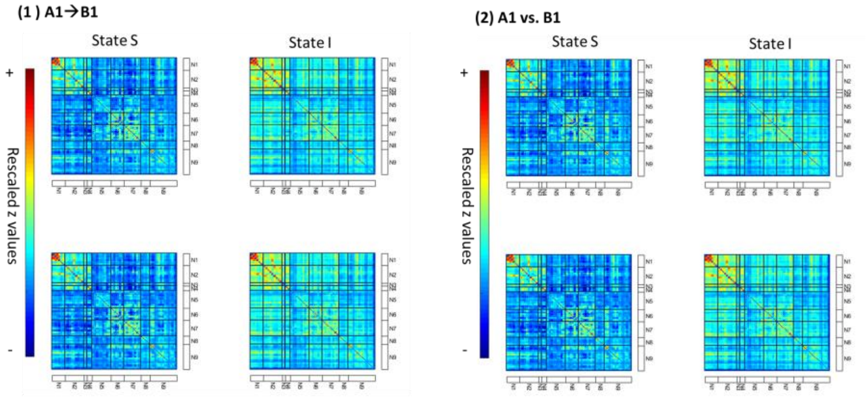

Fig S14. Cluster centroids (k = 5) of the dFC matrices based on 116 ROIs, long, uncentered HCP data. A1→B1: Clustering session A1 and back projection on session B1; A1 vs. B1: Clustering session A1 and B1.

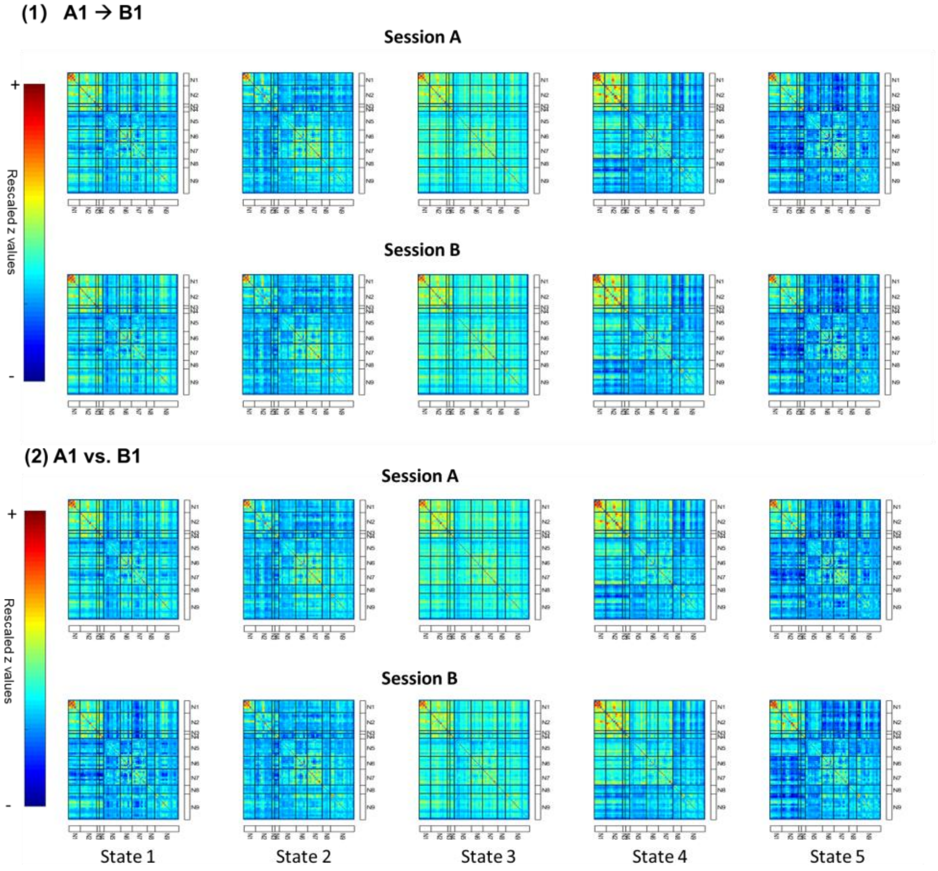

Fig S15. Bar plots of *ICC* values between paired brain states for 116 ROIs, long, uncentered HCP data after removing mean of state centroids. Ses: session; A1→ B1: Clustering session A1 and back projection on session B1; A1 vs. B1: Clustering session A1 and B1 individually.

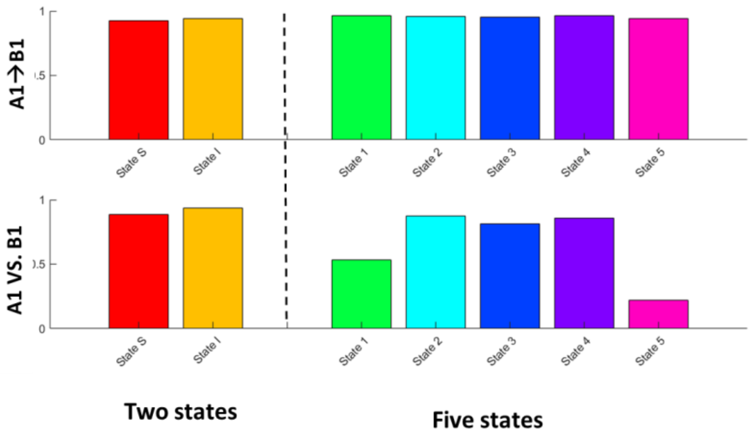

Fig S16. Bar plots of *ICC*s between paired dFC Parameters. Error bar: 95% confidence interval based on bootstrapping with 1000 random samples; A1→ B1: Clustering session A1 and back projection on session B1; A1 vs. B1: Clustering session A1 and B1 individually.

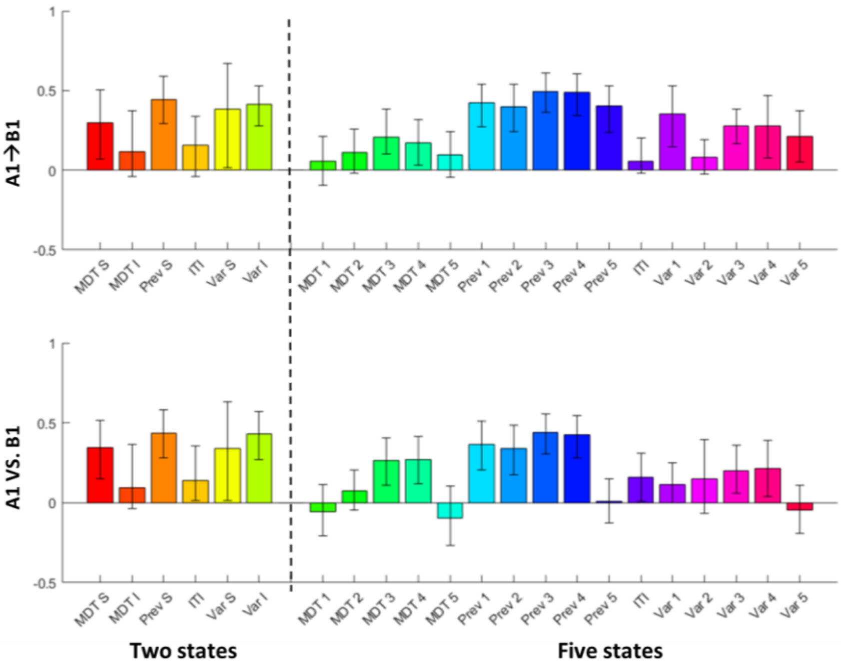

Table S1. Functional network mapping of AAL parcels into nine networks. SM: sensori-motor area; DAN: dorsal attention network; VAN: ventral attention network; FTP: frontal-temporal-parietal; DMN: default mode network.

ROI name	ROI Label	Network
Calcarine_L	43	Visual network
Calcarine_R	44	Visual network
Cuneus_L	45	Visual network
Cuneus_R	46	Visual network
Lingual_L	47	Visual network
Lingual_R	48	Visual network
Occipital_Sup_L	49	Visual network
Occipital_Sup_R	50	Visual network
Occipital_Inf_L	53	Visual network
Occipital_Inf_R	54	Visual network
Fusiform_L	55	Visual network
Fusiform_R	56	Visual network
Precentral_L	1	SM network
Precentral_R	2	SM network
Rolandic_Oper_L	17	SM network
Rolandic_Oper_R	18	SM network
Supp_Motor_Area_L	19	SM network
Supp_Motor_Area_R	20	SM network
Cingulum_Mid_L	33	SM network
Cingulum_Mid_R	34	SM network
Occipital_Mid_L	51	SM network
Occipital_Mid_R	52	SM network
Postcentral_L	57	SM network
Postcentral_R	58	SM network
Paracentral_Lobule_L	69	SM network
Paracentral_Lobule_R	70	SM network
Heschl_L	79	SM network
Heschl_R	80	SM network
Temporal_Sup_L	81	SM network
Temporal_Sup_R	82	SM network
Parietal_Sup_L	59	DAN
Parietal_Sup_R	60	DAN
Insula_L	29	VAN
Insula_R	30	VAN
SupraMarginal_L	63	VAN
SupraMarginal_R	64	VAN
Frontal_Sup_Orb_L	5	Limbic network
Frontal_Sup_Orb_R	6	Limbic network
Olfactory_L	21	Limbic network
Olfactory_R	22	Limbic network
Rectus_L	27	Limbic network
Rectus_R	28	Limbic network
Hippocampus_L	37	Limbic network
Hippocampus_R	38	Limbic network
ParaHippocampal_L	39	Limbic network
ParaHippocampal_R	40	Limbic network
Amygdala_L	41	Limbic network
Amygdala_R	42	Limbic network
Temporal_Pole_Sup_L	83	Limbic network
Temporal_Pole_Sup_R	84	Limbic network
Temporal_Pole_Mid_L	87	Limbic network
Temporal_Pole_Mid_R	88	Limbic network
Temporal_Inf_L	89	Limbic network
Temporal_Inf_R	90	Limbic network
Frontal_Mid_L	7	FTP Cognitive
Frontal_Mid_R	8	FTP Cognitive
Frontal_Mid_Orb_L	9	FTP Cognitive
Frontal_Mid_Orb_R	10	FTP Cognitive
Frontal_Inf_Oper_L	11	FTP Cognitive
Frontal_Inf_Oper_R	12	FTP Cognitive
Frontal_Inf_Tri_L	13	FTP Cognitive
Frontal_Inf_Tri_R	14	FTP Cognitive
Cingulum_Post_L	35	FTP Cognitive
Cingulum_Post_R	36	FTP Cognitive
Parietal_Inf_L	61	FTP Cognitive
Parietal_Inf_R	62	FTP Cognitive
Frontal_Sup_L	3	DMN
Frontal_Sup_R	4	DMN

Frontal_Inf_Orb_L	15	DMN	Cerebelum_Crus2_L	93	Cerebellum
Frontal_Inf_Orb_R	16	DMN	Cerebelum_Crus2_R	94	Cerebellum
Frontal_Sup_Medial_L	23	DMN	Cerebelum_3_L	95	Cerebellum
Frontal_Sup_Medial_R	24	DMN	Cerebelum_3_R	96	Cerebellum
Frontal_Med_Orb_L	25	DMN	Cerebelum_4_5_L	97	Cerebellum
Frontal_Med_Orb_R	26	DMN	Cerebelum_4_5_R	98	Cerebellum
Cingulum_Ant_L	31	DMN	Cerebelum_6_L	99	Cerebellum
Cingulum_Ant_R	32	DMN	Cerebelum_6_R	100	Cerebellum
Angular_L	65	DMN	Cerebelum_7b_L	101	Cerebellum
Angular_R	66	DMN	Cerebelum_7b_R	102	Cerebellum
Precuneus_L	67	DMN	Cerebelum_8_L	103	Cerebellum
Precuneus_R	68	DMN	Cerebelum_8_R	104	Cerebellum
Temporal_Mid_L	85	DMN	Cerebelum_9_L	105	Cerebellum
Temporal_Mid_R	86	DMN	Cerebelum_9_R	106	Cerebellum
Caudate_L	71	Basal gansalia	Cerebelum_10_L	107	Cerebellum
Caudate_R	72	Basal gansalia	Cerebelum_10_R	108	Cerebellum
Putamen_L	73	Basal gansalia	Vermis_1_2	109	Cerebellum
Putamen_R	74	Basal gansalia	Vermis_3	110	Cerebellum
Pallidum_L	75	Basal gansalia	Vermis_4_5	111	Cerebellum
Pallidum_R	76	Basal gansalia	Vermis_6	112	Cerebellum
Thalamus_L	77	Basal gansalia	Vermis_7	113	Cerebellum
Thalamus_R	78	Basal gansalia	Vermis_8	114	Cerebellum
Cerebelum_Crus1_L	91	Cerebellum	Vermis_9	115	Cerebellum
Cerebelum_Crus1_R	92	Cerebellum	Vermis_10	116	Cerebellum

Table S2. Functional network mapping of joint atlas into nine networks. SM: sensori-motor area; DAN: dorsal attention network; VAN: ventral attention network; FTP: frontal-temporal-parietal; DMN: default mode network.

**Region Name**	**ROI Label**	**ROI Name**
Vis_1	1	Visual network
Vis_2	2	Visual network
Vis_3	3	Visual network
Vis_4	4	Visual network
Vis_5	5	Visual network
Vis_6	6	Visual network
Vis_7	7	Visual network
Vis_8	8	Visual network
Vis_9	9	Visual network
Vis_10	10	Visual network
Vis_11	11	Visual network
Vis_12	12	Visual network
Vis_13	13	Visual network
Vis_14	14	Visual network
Vis_15	15	Visual network
Vis_16	16	Visual network
Vis_17	17	Visual network
Vis_18	18	Visual network
Vis_19	19	Visual network
Vis_20	20	Visual network
Vis_21	21	Visual network
Vis_22	22	Visual network
Vis_23	23	Visual network
Vis_24	24	Visual network
Vis_25	25	Visual network
Vis_26	26	Visual network
Vis_27	27	Visual network
Vis_28	28	Visual network
Vis_29	29	Visual network
Vis_30	30	Visual network
Vis_31	31	Visual network
SomMot_1	32	SM network
SomMot_2	33	SM network
SomMot_3	34	SM network
SomMot_4	35	SM network
SomMot_5	36	SM network
SomMot_6	37	SM network
SomMot_7	38	SM network
SomMot_8	39	SM network
SomMot_9	40	SM network
SomMot_10	41	SM network
SomMot_11	42	SM network
SomMot_12	43	SM network
SomMot_13	44	SM network
SomMot_14	45	SM network
SomMot_15	46	SM network
SomMot_16	47	SM network
SomMot_17	48	SM network
SomMot_18	49	SM network
SomMot_19	50	SM network
SomMot_20	51	SM network
SomMot_21	52	SM network
SomMot_22	53	SM network
SomMot_23	54	SM network
SomMot_24	55	SM network
SomMot_25	56	SM network
SomMot_26	57	SM network
SomMot_27	58	SM network
SomMot_28	59	SM network
SomMot_29	60	SM network
SomMot_30	61	SM network
SomMot_31	62	SM network
SomMot_32	63	SM network
SomMot_33	64	SM network
SomMot_34	65	SM network
SomMot_35	66	SM network
SomMot_36	67	SM network
SomMot_37	68	SM network

Post_1	69	DAN
Post_2	70	DAN
Post_3	71	DAN
Post_4	72	DAN
Post_5	73	DAN
Post_6	74	DAN
Post_7	75	DAN
Post_8	76	DAN
Post_9	77	DAN
Post_10	78	DAN
Post_11	79	DAN
Post_12	80	DAN
Post_13	81	DAN
Post_14	82	DAN
Post_15	83	DAN
Post_16	84	DAN
Post_17	85	DAN
FEF_1	86	DAN
FEF_2	87	DAN
FEF_3	88	DAN
FEF_4	89	DAN
PrCv_1	90	DAN
PrCv_2	91	DAN
ParOper_1	92	VAN
ParOper_2	93	VAN
ParOper_3	94	VAN
ParOper_4	95	VAN
TempOcc_1	96	VAN
FrOperIns_1	97	VAN
FrOperIns_2	98	VAN
FrOperIns_3	99	VAN
FrOperIns_4	100	VAN
FrOperIns_5	101	VAN
FrOperIns_6	102	VAN
FrOperIns_7	103	VAN
FrOperIns_8	104	VAN
FrOperIns_9	105	VAN
PFCl_1	106	VAN
Med_1	107	VAN
Med_2	108	VAN
Med_3	109	VAN
Med_4	110	VAN
Med_5	111	VAN
Med_6	112	VAN
Med_7	113	VAN
OFC_1	114	Limbic network
OFC_2	115	Limbic network
OFC_3	116	Limbic network
OFC_4	117	Limbic network
OFC_5	118	Limbic network
TempPole_1	119	Limbic network
TempPole_2	120	Limbic network
TempPole_3	121	Limbic network
TempPole_4	122	Limbic network
TempPole_5	123	Limbic network
TempPole_6	124	Limbic network
TempPole_7	125	Limbic network
TempPole_8	126	Limbic network
Par_1	127	FTP Cognitive
Par_2	128	FTP Cognitive
Par_3	129	FTP Cognitive
Par_4	130	FTP Cognitive
Par_5	131	FTP Cognitive
Par_6	132	FTP Cognitive
Temp_1	133	FTP Cognitive
OFC_1	134	FTP Cognitive
PFCl_1	135	FTP Cognitive
PFCl_2	136	FTP Cognitive
PFCl_3	137	FTP Cognitive
PFCl_4	138	FTP Cognitive
PFCl_5	139	FTP Cognitive
PFCl_6	140	FTP Cognitive
PFCl_7	141	FTP Cognitive
PFCl_8	142	FTP Cognitive
PFCv_1	143	FTP Cognitive
pCun_1	144	FTP Cognitive
pCun_2	145	FTP Cognitive
Cing_1	146	FTP Cognitive
Cing_2	147	FTP Cognitive
PFCmp_1	148	FTP Cognitive

Temp_1	149	DMN	PFC_24	189	DMN
Temp_2	150	DMN	pCunPCC_1	190	DMN
Temp_3	151	DMN	pCunPCC_2	191	DMN
Temp_4	152	DMN	pCunPCC_3	192	DMN
Temp_5	153	DMN	pCunPCC_4	193	DMN
Temp_6	154	DMN	pCunPCC_5	194	DMN
Temp_7	155	DMN	pCunPCC_6	195	DMN
Temp_8	156	DMN	pCunPCC_7	196	DMN
Temp_9	157	DMN	pCunPCC_8	197	DMN
Temp_10	158	DMN	pCunPCC_9	198	DMN
Par_1	159	DMN	pCunPCC_10	199	DMN
Par_2	160	DMN	pCunPCC_11	200	DMN
Par_3	161	DMN	Vis_1	201	Visual network
Par_4	162	DMN	Vis_2	202	Visual network
Par_5	163	DMN	Vis_3	203	Visual network
Par_6	164	DMN	Vis_4	204	Visual network
Par_7	165	DMN	Vis_5	205	Visual network
PFC_1	166	DMN	Vis_6	206	Visual network
PFC_2	167	DMN	Vis_7	207	Visual network
PFC_3	168	DMN	Vis_8	208	Visual network
PFC_4	169	DMN	Vis_9	209	Visual network
PFC_5	170	DMN	Vis_10	210	Visual network
PFC_6	171	DMN	Vis_11	211	Visual network
PFC_7	172	DMN	Vis_12	212	Visual network
PFC_8	173	DMN	Vis_13	213	Visual network
PFC_9	174	DMN	Vis_14	214	Visual network
PFC_10	175	DMN	Vis_15	215	Visual network
PFC_11	176	DMN	Vis_16	216	Visual network
PFC_12	177	DMN	Vis_17	217	Visual network
PFC_13	178	DMN	Vis_18	218	Visual network
PFC_14	179	DMN	Vis_19	219	Visual network
PFC_15	180	DMN	Vis_20	220	Visual network
PFC_16	181	DMN	Vis_21	221	Visual network
PFC_17	182	DMN	Vis_22	222	Visual network
PFC_18	183	DMN	Vis_23	223	Visual network
PFC_19	184	DMN	Vis_24	224	Visual network
PFC_20	185	DMN	Vis_25	225	Visual network
PFC_21	186	DMN	Vis_26	226	Visual network
PFC_22	187	DMN	Vis_27	227	Visual network
PFC_23	188	DMN	Vis_28	228	Visual network

Vis_29	229	Visual network	SomMot_39	269	SM network
Vis_30	230	Visual network	SomMot_40	270	SM network
SomMot_1	231	SM network	Post_1	271	DAN
SomMot_2	232	SM network	Post_2	272	DAN
SomMot_3	233	SM network	Post_3	273	DAN
SomMot_4	234	SM network	Post_4	274	DAN
SomMot_5	235	SM network	Post_5	275	DAN
SomMot_6	236	SM network	Post_6	276	DAN
SomMot_7	237	SM network	Post_7	277	DAN
SomMot_8	238	SM network	Post_8	278	DAN
SomMot_9	239	SM network	Post_9	279	DAN
SomMot_10	240	SM network	Post_10	280	DAN
SomMot_11	241	SM network	Post_11	281	DAN
SomMot_12	242	SM network	Post_12	282	DAN
SomMot_13	243	SM network	Post_13	283	DAN
SomMot_14	244	SM network	Post_14	284	DAN
SomMot_15	245	SM network	Post_15	285	DAN
SomMot_16	246	SM network	Post_16	286	DAN
SomMot_17	247	SM network	Post_17	287	DAN
SomMot_18	248	SM network	Post_18	288	DAN
SomMot_19	249	SM network	Post_19	289	DAN
SomMot_20	250	SM network	FEF_1	290	DAN
SomMot_21	251	SM network	FEF_2	291	DAN
SomMot_22	252	SM network	FEF_3	292	DAN
SomMot_23	253	SM network	PrCv_1	293	DAN
SomMot_24	254	SM network	TempOccPar_1	294	VAN
SomMot_25	255	SM network	TempOccPar_2	295	VAN
SomMot_26	256	SM network	TempOccPar_3	296	VAN
SomMot_27	257	SM network	TempOccPar_4	297	VAN
SomMot_28	258	SM network	TempOccPar_5	298	VAN
SomMot_29	259	SM network	TempOccPar_6	299	VAN
SomMot_30	260	SM network	TempOccPar_7	300	VAN
SomMot_31	261	SM network	PrC_1	301	VAN
SomMot_32	262	SM network	FrOperIns_1	302	VAN
SomMot_33	263	SM network	FrOperIns_2	303	VAN
SomMot_34	264	SM network	FrOperIns_3	304	VAN
SomMot_35	265	SM network	FrOperIns_4	305	VAN
SomMot_36	266	SM network	FrOperIns_5	306	VAN
SomMot_37	267	SM network	FrOperIns_6	307	VAN
SomMot_38	268	SM network	FrOperIns_7	308	VAN

FrOperIns_8	309	VAN	PFCl_9	349	FTP Cognitive
PFCl_1	310	VAN	PFCl_10	350	FTP Cognitive
Med_1	311	VAN	PFCl_11	351	FTP Cognitive
Med_2	312	VAN	PFCl_12	352	FTP Cognitive
Med_3	313	VAN	PFCl_13	353	FTP Cognitive
Med_4	314	VAN	PFCl_14	354	FTP Cognitive
Med_5	315	VAN	PFCl_15	355	FTP Cognitive
Med_6	316	VAN	pCun_1	356	FTP Cognitive
Med_7	317	VAN	pCun_2	357	FTP Cognitive
Med_8	318	VAN	Cing_1	358	FTP Cognitive
OFC_1	319	Limbic network	Cing_2	359	FTP Cognitive
OFC_2	320	Limbic network	PFCmp_1	360	FTP Cognitive
OFC_3	321	Limbic network	PFCmp_2	361	FTP Cognitive
OFC_4	322	Limbic network	Par_1	362	DMN
OFC_5	323	Limbic network	Par_2	363	DMN
OFC_6	324	Limbic network	Par_3	364	DMN
TempPole_1	325	Limbic network	Par_4	365	DMN
TempPole_2	326	Limbic network	Par_5	366	DMN
TempPole_3	327	Limbic network	Temp_1	367	DMN
TempPole_4	328	Limbic network	Temp_2	368	DMN
TempPole_5	329	Limbic network	Temp_3	369	DMN
TempPole_6	330	Limbic network	Temp_4	370	DMN
TempPole_7	331	Limbic network	Temp_5	371	DMN
Par_1	332	FTP Cognitive	Temp_6	372	DMN
Par_2	333	FTP Cognitive	Temp_7	373	DMN
Par_3	334	FTP Cognitive	Temp_8	374	DMN
Par_4	335	FTP Cognitive	PFCv_1	375	DMN
Par_5	336	FTP Cognitive	PFCv_2	376	DMN
Par_6	337	FTP Cognitive	PFCv_3	377	DMN
Temp_1	338	FTP Cognitive	PFCv_4	378	DMN
Temp_2	339	FTP Cognitive	PFCdPFCm_1	379	DMN
PFCv_1	340	FTP Cognitive	PFCdPFCm_2	380	DMN
PFCl_1	341	FTP Cognitive	PFCdPFCm_3	381	DMN
PFCl_2	342	FTP Cognitive	PFCdPFCm_4	382	DMN
PFCl_3	343	FTP Cognitive	PFCdPFCm_5	383	DMN
PFCl_4	344	FTP Cognitive	PFCdPFCm_6	384	DMN
PFCl_5	345	FTP Cognitive	PFCdPFCm_7	385	DMN
PFCl_6	346	FTP Cognitive	PFCdPFCm_8	386	DMN
PFCl_7	347	FTP Cognitive	PFCdPFCm_9	387	DMN
PFCl_8	348	FTP Cognitive	PFCdPFCm_10	388	DMN

PFCdPFCm_11	389	DMN	Right_I_IV	2	Cerebellum
PFCdPFCm_12	390	DMN	Left_V	3	Cerebellum
PFCdPFCm_13	391	DMN	Right_V	4	Cerebellum
pCunPCC_1	392	DMN	Left_VI	5	Cerebellum
pCunPCC_2	393	DMN	Vermis_VI	6	Cerebellum
pCunPCC_3	394	DMN	Right_VI	7	Cerebellum
pCunPCC_4	395	DMN	Left_CrusI	8	Cerebellum
pCunPCC_5	396	DMN	Vermis_CrusI	9	Cerebellum
pCunPCC_6	397	DMN	Right_CrusI	10	Cerebellum
pCunPCC_7	398	DMN	Left_CrusII	11	Cerebellum
pCunPCC_8	399	DMN	Vermis_CrusII	12	Cerebellum
pCunPCC_9	400	DMN	Right_CrusII	13	Cerebellum
Thalamus_L	4	Basal gansalia	Left_VIIb	14	Cerebellum
Caudate_L	5	Basal gansalia	Vermis_VIIb	15	Cerebellum
Putamen_L	6	Basal gansalia	Right_VIIb	16	Cerebellum
Pallidum_L	7	Basal gansalia	Left_VIIIa	17	Cerebellum
Hippocampus_L	9	Basal gansalia	Vermis_VIIIa	18	Cerebellum
Amygdala_L	10	Basal gansalia	Right_VIIIa	19	Cerebellum
Accumbens_L	11	Basal gansalia	Left_VIIIb	20	Cerebellum
Thalamus_R	15	Basal gansalia	Vermis_VIIIb	21	Cerebellum
Caudate_R	16	Basal gansalia	Right_VIIIb	22	Cerebellum
Putamen_R	17	Basal gansalia	Left_IX	23	Cerebellum
Pallidum_R	18	Basal gansalia	Vermis_IX	24	Cerebellum
Hippocampus_R	19	Basal gansalia	Right_IX	25	Cerebellum
Amygdala_R	20	Basal gansalia	Left_X	26	Cerebellum
Accumbens_R	21	Basal gansalia	Vermis_X	27	Cerebellum
Left_I_IV	1	Cerebellum	Right_X	28	Cerebellum

4

Table S3. Information about overlapping voxels between different atlases used for the joint-442-ROI atlas. Note that time courses were extracted from regions of interest (ROIs) as defined by each atlas without correcting for overlap. HO: Harvard-Oxford subcortical atlas; SUIT: spatially unbiased infra-tentorial template.

ROI label in HO	Voxel number in HO	ROI label in Schaefer	Voxel number in Schaefer atlas	Voxel number of overlap
4	1470	13	119	1
9	765	2	425	8
9	765	7	183	2
9	765	20	120	2
9	765	126	203	7
10	332	125	431	2
11	89	117	405	2
15	1401	214	217	1
19	765	202	348	2
19	765	214	217	1
19	765	217	127	1
19	765	328	481	1
19	765	331	279	9
20	413	329	471	12
21	84	323	309	1
**Subcortical HO & cerebellum SUIT atlases**				
**ROI label in HO**	**Voxel number in HO**	**ROI label in SUIT**	**Voxel number in SUIT**	**Voxel number of overlap**
4	1470	1	935	1
**Cerebellum SUIT & cortex Schaefer atlases**				
**ROI label in HO**	**Voxel number in HO**	**ROI label in Schaefer**	**Voxel number in Schaefer**	**Voxel number of overlap**
1	935	2	425	3
1	935	7	183	19
1	935	10	261	3
1	935	13	119	18
1	935	126	203	5
2	1004	201	363	9
2	1004	202	348	11
2	1004	207	136	22
2	1004	212	348	15
2	1004	214	217	4
2	1004	331	279	3
3	978	1	347	51
3	978	2	425	24

3	978	4	401	12
3	978	7	183	21
3	978	10	261	54
3	978	13	119	10
3	978	126	203	2
4	932	201	363	69
4	932	202	348	5
4	932	203	355	8
4	932	205	453	26
4	932	207	136	11
4	932	211	328	2
4	932	212	348	57
5	1915	1	347	85
5	1915	3	309	40
5	1915	4	401	19
5	1915	5	426	8
5	1915	9	320	5
5	1915	10	261	10
5	1915	11	308	8
5	1915	69	363	9
7	1772	201	363	33
7	1772	203	355	75
7	1772	204	386	9
7	1772	205	453	13
7	1772	206	530	35
7	1772	208	279	1
7	1772	211	328	24
7	1772	271	738	1
8	2716	3	309	4
8	2716	69	363	25
8	2716	70	612	9
10	2767	203	355	2
10	2767	204	386	9
10	2767	208	279	2
10	2767	215	719	1
10	2767	271	738	13
13	1998	330	434	1

Table S4. Available subjects for the parameters of mean dwell time (*MDT*) for states of segregation (S) and integration (I) and inter-transition interval (*ITI*).

Centering	Time length	ROIs	Session	*MDT S*	*MDT I*	*ITI*
**OWN data (23 subjects)**						
No	16'27"	116	A	20	20	20
	16'27"	116	B	22	21	21
No	8'14"	116	A	19	20	19
	8'14"	116	B	21	22	21
No	16'27"	442	A	20	20	20
	16'27"	442	B	21	22	21
No	8'14"	442	A	16	17	15
	8'14"	442	B	21	20	20
Yes	16'27"	116	A	23	23	23
	16'27"	116	B	23	23	23
Yes	8'14"	116	A	23	23	23
	8'14"	116	B	23	23	23
Yes	16'27"	442	A	23	23	23
	16'27"	442	B	23	23	23
Yes	8'14"	442	A	23	22	22
	8'14"	442	B	23	23	23
**HCP data (501 subjects)**						
No	14'24"	116	A	492	485	492
	14'24"	116	B	493	482	493
No	7'12"	116	A	473	447	473
	7'12"	116	B	469	452	469
No	14'24"	442	A	489	491	489
	14'24"	442	B	487	482	487
No	7'12"	442	A	458	449	458
	7'12"	442	B	449	446	449
Yes	14'24"	116	A	501	501	501
	14'24"	116	B	501	501	501
Yes	7'12"	116	A	497	500	497
	7'12"	116	B	498	497	498
Yes	14'24"	442	A	501	501	501
	14'24"	442	B	501	501	501
Yes	7'12"	442	A	498	500	498
	7'12"	442	B	497	495	497

Table S5. Reliability (measured by Cosine distance) between centroids of the two clusters for the two sessions. Ses: session**; S:** segregated state**; I:** integrated state.

		16'27'' OWN data		14'24'' HCP data		8'14'' OWN data		7'12'' HCP data	
**116 ROIs**									
**a)**	**Clusters based on uncentered data**								
		**Ses A**		**Ses A**		**Ses A**		**Ses A**	
		S	I	S	I	S	I	S	I
**Ses B**	S	0.01	0.09	0.00	0.15	0.02	0.07	0.00	0.13
	I	0.11	0.00	0.16	0.00	0.11	0.01	0.14	0.00
**b)**	**Clusters based on centered data**								
		**Ses A**		**Ses A**		**Ses A**		**Ses A**	
		S	I	S	I	S	I	S	I
**Ses B**	S	0.01	1.99	0.00	2.00	0.03	1.97	0.01	1.99
	I	1.99	0.01	2.00	0.00	1.97	0.03	1.99	0.01
**c)**	**Clusters based on uncentered data & removing mean of cluster centroid**								
		**Ses A**		**Ses A**		**Ses A**		**Ses A**	
		S	I	S	I	S	I	S	I
**Ses B**	S	0.16	1.95	0.01	1.99	0.37	1.81	0.01	2.00
	I	1.96	0.08	1.99	0.01	1.87	0.23	2.00	0.01
**442 ROIs**									
**d)**	**Clusters based on uncentered data**								
		**Ses A**		**Ses A**		**Ses A**		**Ses A**	
		S	I	S	I	S	I	S	I
**Ses B**	S	0.02	0.13	0.00	0.16	0.04	0.13	0.00	0.12
	I	0.14	0.00	0.17	0.00	0.14	0.01	0.13	0.00
**e)**	**Clusters based on centered data**								
		**Ses A**		**Ses A**		**Ses A**		**Ses A**	
		S	I	S	I	S	I	S	I
**Ses B**	S	0.02	1.98	0.01	1.99	0.04	1.96	0.01	1.99
	I	1.98	0.02	1.99	0.01	1.96	0.04	1.98	0.01
**f)**	**Clusters based on uncentered data & removing mean of cluster centroid**								
		**Ses A**		**Ses A**		**Ses A**		**Ses A**	
		S	I	S	I	S	I	S	I
**Ses B**	S	0.14	1.96	0.01	2.00	0.31	1.90	0.01	2.00
	I	1.96	0.06	2.00	0.01	1.91	0.14	2.00	0.01

Table S6. Graph theoretical measures of clustering results based on 16'27" data. Btsp.: bootstrapping; C.I.: 95% confidence interval; ses.: session.

Uncentered				Centered (adding mean back)		
**AAL atlas**						
**State S**	Btsp. C.I. of ses. A	ses. A	ses. B	Btsp. C.I. of ses. A	ses. A	ses. B
global efficiency	[0.3242, 0.3588]	0.3354	0.3449	[0.3243, 0.3725]	0.3425	0.3486
modularity	[0.1396, 0.1850]	0.1553	0.1779	[0.1157, 0.1598]	0.1358	0.1245
**State I**						
global efficiency	[0.4819, 0.5455]	0.5092	0.5033	[0.5062, 0.5972]	0.5473	0.5536
modularity	[0.0583, 0.0826]	0.0704	0.0778	[0.0539, 0.0740]	0.0637	0.0659
**Joint atlas**						
**State S**	Btsp. C.I. of ses. A	ses. A	ses. B	Btsp. C.I. of ses. A	ses. A	ses. B
global efficiency	[0.2868, 0.3214]	0.2948	0.3039	[0.2956, 0.3464]	0.3125	0.3191
modularity	[0.1770, 0.2060]	0.1896	0.2003	[0.1218, 0.1658]	0.1433	0.1362
**State I**						
global efficiency	[0.4420, 0.4965]	0.4645	0.4603	[0.4736, 0.5696]	0.5170	0.5225
modularity	[0.0587, 0.0715]	0.0641	0.0707	[0.0511, 0.0694]	0.0581	0.0576

Table S7. Reliability of dFC parameters in terms of Spearman’s correlation. CI: 95% confidence interval based on bootstrapping (1000 times). ***MDT***: Mean dwell time; ***Prev***: Prevalence; **ITI**: Inter-transition interval; ***Var***: variability; **S**: segregated state; **I**: integrated state. Bold: confidence interval not including zero.

		**16’27’’ OWN data**		**14'24'' HCP data**		**8’14’’ OWN data**		**7'12'' HCP data**	
					**116 ROIs**				
		**Uncentered**	**Centered**	**Uncentered**	**Centered**	**Uncentered**	**Centered**	**Uncentered**	**Centered**
MDT	**S**	0.22 [-0.26, 0.64]	0.05 [-0.51, 0.53]	**0.25 [0.16, 0.34]**	**0.11 [0.02, 0.19]**	0.41 [-0.55, 0.97]	0.30 [-0.13, 0.70]	**0.14 [0.04, 0.24]**	0.04 [-0.04, 0.12]
	**I**	0.16 [-0.35, 0.60]	0.22 [-0.29, 0.63]	**0.27 [0.19, 0.36]**	0.06 [-0.03, 0.15]	0.02 [-0.83, 0.63]	-0.00 [-0.44, 0.44]	**0.11 [0.02, 0.21]**	**0.11 [0.02, 0.20]**
Prev	**S**	**0.55 [0.15, 0.81]**	0.08 [-0.44, 0.50]	**0.46 [0.38, 0.53]**	-0.00 [-0.08, 0.08]	0.37 [-0.01, 0.69]	0.20 [-0.23, 0.60]	**0.39 [0.31, 0.46]**	0.06 [-0.03, 0.14]
ITI		-0.09 [-0.54, 0.40]	0.09 [-0.41, 0.57]	0.03 [-0.06, 0.12]	**0.10 [0.02, 0.19]**	-0.21 [-0.96, 0.81]	0.29 [-0.18, 0.69]	0.04 [-0.05, 0.14]	0.03 [-0.06, 0.13]
Var	**S**	**0.44 [0.03, 0.75]**	0.27 [-0.22, 0.71]	**0.31 [0.22, 0.40]**	**0.22 [0.12, 0.31]**	0.00 [-0.48, 0.50]	-0.05 [-0.49, 0.43]	**0.32 [0.23, 0.40]**	**0.09 [0.01, 0.18]**
	**I**	**0.43 [0.08, 0.68]**	0.20 [-0.23, 0.55]	**0.36 [0.28, 0.44]**	**0.21 [0.12, 0.29]**	-0.08 [-0.57, 0.45]	-0.21 [-0.68, 0.29]	**0.29 [0.20, 0.37]**	0.06 [-0.04, 0.16]
					**442 ROIs**				
MDT	**S**	-0.37 [-0.66, 0.10]	-0.13 [-0.57, 0.33]	**0.28 [0.19, 0.36]**	0.07 [-0.01, 0.15]	-0.25 [-0.72, 0.34]	0.39 [-0.05, 0.76]	**0.14 [0.04, 0.24]**	0.01 [-0.07, 0.09]
	**I**	-0.21 [-0.79, 0.37]	0.34 [-0.03, 0.61]	**0.29 [0.20, 0.37]**	**0.11 [0.01, 0.20]**	0.13 [-0.44, 0.66]	0.34 [-0.16, 0.71]	**0.16 [0.06, 0.25]**	**0.10 [0.01, 0.18]**
Prev	**S**	**0.43 [-0.07, 0.76]**	0.32 [-0.10, 0.69]	**0.48 [0.40, 0.56]**	0.04 [-0.04, 0.12]	0.31 [-0.09, 0.66]	-0.04 [-0.48, 0.42]	**0.42 [0.34, 0.49]**	0.07 [-0.02, 0.17]
ITI		-0.18 [-0.60, 0.30]	-0.33 [-0.72, 0.17]	**0.10 [0.01, 0.20]**	**0.12 [0.03, 0.20]**	0.03 [-0.53, 0.54]	0.25 [-0.25, 0.69]	0.05 [-0.05, 0.15]	0.06 [-0.03, 0.16]
Var	**S**	0.23 [-0.27, 0.67]	0.26 [-0.19, 0.63]	**0.35 [0.27, 0.43]**	**0.29 [0.20, 0.37]**	0.26 [-0.31, 0.71]	-0.03 [-0.44, 0.41]	**0.36 [0.28, 0.45]**	**0.11 [0.02, 0.19]**
	**I**	0.33 [-0.09, 0.64]	0.10 [-0.33, 0.50]	**0.41 [0.32, 0.48]**	**0.24 [0.16, 0.32]**	0.38 [-0.03, 0.69]	-0.05 [-0.49, 0.48]	**0.32 [0.24, 0.40]**	**0.15 [0.06, 0.24]**

Table S8. Values of mean (with standard error of mean) for dynamic parameters across all the strategies for uncentered data. ***MDT***: Mean dwell time; ***Prev***: Prevalence; **ITI**: Inter-transition interval; ***Var***: variability; Ses: session; **S**: segregated state; **I**: integrated state.

	16'27" OWN data		14'24" HCP data		8'14" OWN data		7'12" HCP data	
	Ses A	Ses B	Ses A	Ses B	Ses A	Ses B	Ses A	Ses B
**Uncentered (116 ROIs)**								
**MDT S**	56.8395<br>(9.5531)	120.3119<br>(42.4152)	45.4065<br>(1.8137)	47.6414<br>(1.9734)	54.9265<br>(14.5383)	69.0426<br>(18.3573)	42.5703<br>(1.9361)	43.6906<br>(1.8681)
**MDT I**	62.7101<br>(10.6197)	55.3948<br>(10.0364)	63.9379<br>(2.8942)	59.6142<br>(2.8287)	63.2338<br>(15.9930)	42.7176<br>(14.2708)	47.6312<br>(2.0886)	43.3018<br>(1.8402)
Prev	0.4099<br>(0.0667)	0.5006<br>(0.0645)	0.4166<br>(0.0115)	0.4242<br>(0.0115)	0.4923<br>(0.0867)	0.5929<br>(0.0771)	0.4283<br>(0.0120)	0.4377<br>(0.0121)
**ITI**	59.7748<br>(5.5877)	90.6474<br>(21.6195)	54.1235<br>(1.3677)	53.7626<br>(1.5369)	60.5480<br>(10.3510)	57.6237<br>(10.1598)	44.3740<br>(1.2361)	43.7049<br>(1.2753)
***Var* S**	47.2801<br>(0.9947)	44.7765<br>(2.5836)	46.8278<br>(0.2498)	46.6184<br>(0.2985)	44.5663<br>(1.6487)	43.4735<br>(2.3145)	42.4204<br>(0.3924)	42.8596<br>(0.3875)
***Var* I**	47.5835<br>(0.9381)	46.9340<br>(1.2673)	48.6086<br>(0.1450)	48.2532<br>(0.1910)	44.7412<br>(2.0064)	39.1420<br>(3.8110)	45.2675<br>(0.2597)	45.0241<br>(0.2402)
**Uncentered (442 ROIs)**								
**MDT S**	51.0424<br>(8.6506)	54.1497<br>(7.2686)	58.0331<br>(3.0160)	59.9289<br>(3.0885)	68.4518<br>(23.2948)	58.2041<br>(8.0348)	52.3661<br>(2.3233)	52.2068<br>(2.5788)
**MDT I**	85.8781<br>(37.8671)	49.8348<br>(12.3163)	62.4226<br>(3.1167)	59.6254<br>(2.5631)	59.1686<br>(11.5504)	43.4480<br>(10.1016)	42.6415<br>(1.9389)	41.8627<br>(1.9442)
Prev	0.3719<br>(0.0615)	0.4710<br>(0.0595)	0.4819<br>(0.0122)	0.4857<br>(0.0122)	0.3669<br>(0.0710)	0.5171<br>(0.0615)	0.5176<br>(0.0128)	0.5259<br>(0.0131)
**ITI**	68.4603<br>(19.8071)	51.9922<br>(5.8950)	60.4667<br>(1.8909)	60.0059<br>(1.7825)	62.5486<br>(11.1751)	50.8260<br>(5.9643)	47.4453<br>(1.3876)	46.5458<br>(1.4943)
***Var* S**	173.1534<br>(9.0137)	179.1828<br>(8.0468)	183.9791<br>(0.8191)	182.8877<br>(0.9953)	167.0259<br>(6.7518)	163.9534<br>(10.3244)	169.2963<br>(1.3446)	170.8245<br>(1.2562)
***Var* I**	189.6695<br>(2.5539)	188.5468<br>(3.1962)	184.3452<br>(0.9789)	183.3121<br>(1.0002)	174.6023<br>(5.3733)	174.3634<br>(4.1227)	168.0465<br>(1.3333)	166.4797<br>(1.4495)

Table S9. Values of mean (with standard error of mean) for dynamic parameters across all the strategies for centered data. ***MDT***: Mean dwell time; ***Prev***: Prevalence; **ITI**: Inter-transition interval; ***Var***: variability; Ses: session; **S**: segregated state; **I**: integrated state.

	**16'27" OWN data**		**14'24" HCP data**		**8'14" OWN data**		**7'12" HCP data**	
	**Ses A**	**Ses B**	**Ses A**	**Ses B**	**Ses A**	**Ses B**	**Ses A**	**Ses B**
**Centered (116 ROIs)**								
***MDT* S**	45.2642 (2.5311)	43.6423 (3.7801)	**47.1938 (0.8479)**	48.2696 (0.9690)	47.8416 (7.2430)	47.0381 (6.0814)	45.8579 (1.2138)	45.4690 (1.3253)
***MDT* I**	44.5569 (1.8497)	39.5118 (2.4707)	40.9735 (0.6426)	41.1302 (0.6806)	45.2922 (3.7719)	38.5992 (3.3510)	38.6785 (0.9330)	38.3324 (1.0509)
Prev	0.5250 (0.0086)	0.5455 (0.0128)	0.5461 (0.0023)	0.5496 (0.0024)	0.5303 (0.0139)	0.5295 (0.0153)	0.5462 (0.0030)	0.5444 (0.0031)
ITI	44.9105 (1.7611)	41.5771 (2.6303)	44.0837 (0.6399)	44.6999 (0.6813)	46.5669 (4.7292)	42.8186 (4.3115)	41.9670 (0.9002)	41.1552 (0.9490)
***Var* S**	48.7687 (0.3558)	49.2651 (0.4142)	48.9812 (0.0565)	49.0128 (0.0555)	45.9627 (0.4314)	46.7930 (0.4897)	46.3735 (0.0620)	46.3783 (0.0637)
***Var* I**	46.8177 (0.4605)	47.1901 (0.4123)	47.6304 (0.0767)	47.4897 (0.0804)	44.0797 (0.4977)	45.3066 (0.4050)	44.8841 (0.0871)	44.8052 (0.0870)
**Centered (442 ROIs)**								
***MDT* S**	45.5681 (4.1784)	50.6074 (6.4157)	50.1301 (1.1346)	51.5976 (1.1529)	49.7624 (6.2552)	47.1022 (8.2999)	48.4608 (1.3749)	48.6850 (1.5063)
***MDT* I**	44.6989 (2.8354)	43.1606 (3.7600)	42.3769 (0.7279)	42.4446 (0.7335)	50.1912 (5.8020)	40.3645 (3.7241)	39.2986 (0.9413)	38.5747 (0.9693)
Prev	0.5383 (0.0087)	0.5568 (0.0124)	0.5588 (0.0025)	0.5625 (0.0025)	0.5461 (0.0140)	0.5341 (0.0142)	0.5597 (0.0031)	0.5565 (0.0031)
ITI	45.1335 (3.0241)	46.8840 (4.7985)	46.2535 (0.8119)	47.0211 (0.7883)	47.5284 (4.1759)	43.7333 (5.4817)	43.6716 (0.9489)	42.3682 (0.9067)
***Var* S**	190.8506 (1.4218)	191.8506 (1.5844)	189.8796 (0.2172)	190.0916 (0.2107)	180.9150 (1.4609)	181.3261 (1.6333)	179.7692 (0.2145)	179.9211 (0.2287)
***Var* I**	182.9784 (1.6204)	184.8166 (1.5700)	185.4867 (0.2880)	184.9646 (0.2965)	172.0074 (1.6525)	176.2201 (1.5509)	174.2846 (0.3357)	173.9610 (0.3241)

Table S10. Spearman's correlations between dFC parameters (mean of both sessions) for uncentered data and 116 ROIs (AAL atlas). ***MDT***: Mean dwell time; ***Prev***: Prevalence; ***ITI***: Inter-transition interval; ***Var***: variability; S: state S; I: state I; *: $p < 0.05$; **: $p < 0.001$.

		MDT		***Prev***	***ITI***	***Var***		***MDT***		***Prev***	***ITI***	***Var***	
		**16'27'' OWN data**						**14'24'' HCP data**					
		**S**	**I**	**S**		**S**	**I**	**S**	**I**	**S**		**S**	**I**
MDT	**S**	-	-	-	-	-	-	-	-	-	-	-	-
	**I**	-0.38	-	-	-	-	-	-0.53**	-	-	-	-	-
Prev	**S**	0.77**	-0.76**	-	-	-	-	0.80**	-0.81**	-	-	-	-
ITI		0.68*	0.31	0.17	-	-	-	0.30**	0.52**	-0.12*	-	-	-
Var	**S**	0.42	-0.46*	0.61*	0.08	-	-	0.61**	-0.62**	0.78**	-0.12*	-	-
	**I**	-0.60*	0.58*	-0.72**	-0.020	-0.22	-	-0.54**	0.58**	-0.69**	0.04	-0.40**	-
		**8'14'' OWN data**						**7'12'' HCP data**					
MDT	**S**	-	-	-	-	-	-	-	-	-	-	-	-
	**I**	-0.21	-	-	-	-	-	-0.19*	-	-	-	-	-
Prev	**S**	0.89*	-0.54	-	-	-	-	0.73**	-0.47**	-	-	-	-
ITI		0.57	0.64	0.25	-	-	-	0.58**	0.61**	0.18*	-	-	-
Var	**S**	0.54	-0.50	0.57	0.07	-	-	0.60**	-0.42**	0.86**	0.12	-	-
	**I**	-0.57	0.43	-0.79*	-0.14	-0.50	-	-0.58**	0.22*	-0.75**	-0.27*	-0.56**	-

Table S11. Spearman's correlations between dFC parameters (mean of both sessions) for centered data and 116 ROIs (AAL atlas). *MDT*: Mean dwell time; *Prev*: Prevalence; *ITI*: Inter-transition interval; *Var*: variability; S: state S; I: state I; *: $p < 0.05$; **: $p < 0.001$.

		MDT		***Prev***	***ITI***	***Var***		***MDT***		***Prev***	***ITI***	***Var***	
		**16'27'' OWN data**						**14'24'' HCP data**					
		**S**	**I**	**S**		**S**	**I**	**S**	**I**	**S**		**S**	**I**
MDT	**S**	-	-	-	-	-	-	-	-	-	-	-	-
	**I**	0.30	-	-	-	-	-	0.44**	-	-	-	-	-
Prev	**S**	0.56*	0.00	-	-	-	-	0.41**	-0.19**	-	-	-	-
ITI		0.80**	0.72**	0.37	-	-	-	0.89**	0.77**	0.19**	-	-	-
Var	**S**	-0.06	0.46*	0.21	0.22	-	-	0.15**	0.10*	0.13*	0.16**	-	-
	**I**	0.00	0.20	0.27	0.10	0.61*	-	0.18**	-0.14*	0.31**	0.06	0.56**	-
		**8'14'' OWN data**						**7'12'' HCP data**					
MDT	**S**	-	-	-	-	-	-	-	-	-	-	-	-
	**I**	0.58*	-	-	-	-	-	0.40**	-	-	-	-	-
Prev	**S**	0.67**	0.20	-	-	-	-	0.39**	-0.15	-	-	-	-
ITI		0.94**	0.80**	0.59*	-	-	-	0.89**	0.73**	0.22*	-	-	-
Var	**S**	0.09	0.14	0.15	0.11	-	-	0.18*	0.01	0.26**	0.14	-	-
	**I**	-0.01	-0.22	0.17	-0.09	0.29	-	-0.05	-0.17*	0.05	-0.15	0.38**	-

Table S12. Spearman's correlations between dFC parameters for uncentered data and 442 ROIs. ***MDT***: Mean dwell time; ***Prev***: Prevalence; ***ITI***: Inter-transition interval; ***Var***: variability; **S**: segregated state; **I**: integrated state; *: $p < 0.05$; **: $p < 0.001$.

		MDT		***Prev***	***ITI***	***Var***		***MDT***		***Prev***	***ITI***	***Var***	
		**S**	**I**	**S**		**S**	**I**	**S**	**I**	**S**		**S**	**I**
		**16'27'' OWN data**						**14'24'' HCP data**					
MDT	**S**	-	-	-	-	-	-	-	-	-	-	-	-
	**I**	-0.45	-	-	-	-	-	-0.52**	-	-	-	-	-
Prev	**S**	0.58*	-0.79**	-	-	-	-	0.79**	-0.83**	-	-	-	-
ITI		0.58*	0.39	-0.15	-	-	-	0.36**	0.46**	-0.09	-	-	-
Var	**S**	0.37	-0.46*	0.48*	-0.09	-	-	0.56**	-0.61**	0.72**	-0.12*	-	-
	**I**	-0.50*	0.53*	-0.74**	-0.07	-0.01	-	-0.58**	0.65**	-0.76**	0.04	-0.35**	-
		**8'14'' OWN data**						**7'12'' HCP data**					
MDT	**S**	-	-	-	-	-	-	-	-	-	-	-	-
	**I**	-0.38	-	-	-	-	-	-0.11	-	-	-	-	-
Prev	**S**	0.47	-0.66*	-	-	-	-	0.57**	-0.60**	-	-	-	-
ITI		0.76*	0.17	0.11	-	-	-	0.73**	0.48**	0.13	-	-	-
Var	**S**	0.03	-0.55*	0.73*	-0.36	-	-	0.50**	-0.57**	0.87**	0.05	-	-
	**I**	-0.49	0.41	-0.74*	-0.43	-0.23	-	-0.42**	0.43**	-0.84**	-0.14	-0.62**	-

Table S13. Spearman's correlations between dFC parameters for centered data and 442 ROIs. ***MDT***: Mean dwell time; ***Prev***: Prevalence; ***ITI***: Inter-transition interval; ***Var***: variability; **S**: segregated state; **I**: integrated state; *: $p < 0.05$; **: $p < 0.001$.

		MDT		***Prev***	***ITI***	***Var***		***MDT***		***Prev***	***ITI***	***Var***	
		**S**	**I**	**S**		**S**	**I**	**S**	**I**	**S**		**S**	**I**
		**16'27'' OWN data**						**14'24'' HCP data**					
MDT	**S**	-	-	-	-	-	-	-	-	-	-	-	-
	**I**	0.41	-	-	-	-	-	0.42**	-	-	-	-	-
Prev	**S**	0.19	-0.3	-	-	-	-	0.51**	-0.11*	-	-	-	-
ITI		0.89**	0.74**	0.09	-	-	-	0.89**	0.77**	0.31**	-	-	-
Var	**S**	-0.08	0.59*	-0.39	0.18	-	-	0.09*	0.09*	0.07	0.11*	-	-
	**I**	0.01	0.34	-0.12	0.16	0.58*	-	0.10*	-0.12*	0.21**	0.01	0.60**	-
		**8'14'' OWN data**						**7'12'' HCP data**					
MDT	**S**	-	-	-	-	-	-	-	-	-	-	-	-
	**I**	0.44*	-	-	-	-	-	0.38**	-	-	-	-	-
Prev	**S**	0.69**	0.3	-	-	-	-	0.41**	-0.07	-	-	-	-
ITI		0.88**	0.76**	0.60*	-	-	-	0.84**	0.78**	0.25*	-	-	-
Var	**S**	-0.02	0.21	-0.08	0.11	-	-	0.06	-0.09	0.19*	0.01	-	-
	**I**	0.16	-0.1	0.25	0.02	0.50*	-	-0.09	-0.14	-0.08	-0.16*	0.43**	-

Table S14. Reliability [measured by *ICC* and cosine distance (*CD*)] with global signal regression (GSR) for the two clusters between sessions A and B (backprojection) using 116 ROIs in the long HCP data. Ses: session; S: segregated state; I: integrated state.

		ICC values - Ses. A		*CD* - Ses. A	
		**S**	**I**	**S**	**I**
**a) Clusters based on uncentered data**					
**Ses. B**	**S**	0.99	0.90	0.01	0.10
	**I**	0.95	0.99	0.05	0.06
**b) Clusters based on uncentered data & removing mean of cluster centroid**					
**Ses. B**	**S**	0.75	-0.89	0.20	1.93
	**I**	-0.75	0.89	1.80	0.07

Table S15. Reliability for dFC parameters computed after global signal regression (GSR) using 116 ROIs in the long HCP data. *: $p_{uncorrected} < 0.05$; **: $p_{uncorrected} < 0.001$; ***MDT***: Mean dwell time; ***Prev***: Prevalence; ***ITI***: Inter-transition interval; ***Var***: variability; S: segregated state; I: integrated state.

	MDT S	***MDT***	***Prev S***	***ITI***	***Var S***	***Var I***
ICC	0.25 **<br>[0.1411, 0.3713]	0.12*<br>[0.0360, 0.2274]	0.50**<br>[0.4338, 0.5735]	0.02*<br>[-0.0642, 0.1284]	0.37**<br>[0.2507, 0.4728]	0.18**<br>[0.0806, 0.3087]
rho	0.31**<br>[0.2244, 0.3935]	0.25**<br>[0.1647, 0.3421]	0.51**<br>[0.4380, 0.5737]	0.01<br>[-0.0843, 0.1036]	0.49**<br>[0.4058, 0.5620]	0.25**<br>[0.1612, 0.3297]

Table S16. Reproducibility with 95% confidence intervals (measured by *ICC* and Spearman correlation *rho*) for the same dFC parameters with global signal regression (GSR) and without using 116 ROIs and the long HCP data. *: $p_{uncorrected} < 0.05$; **: $p_{uncorrected} < 0.001$; ***MDT***: Mean dwell time; ***Prev***: Prevalence; ***ITI***: Inter-transition interval; ***Var***: variability; **S**: segregated state; **I**: integrated state.

	MDT S	***MDT I***	***Prev S***	***ITI***	***Var S***	***Var I***
			**Session A**			
ICC	0.27** [0.1812, 0.3847]	0.17** [0.0970, 0.2698]	0.53** [0.4715, 0.5919]	0.08 [-0.0023, 0.1581]	0.18** [0.1242, 0.2512]	0.31** [0.1831, 0.4702]
rho	0.43** [0.3477, 0.5052]	0.35** [0.2579, 0.4391]	0.61** [0.5424, 0.6604]	0.09 [-0.0135, 0.1799]	0.44** [0.3627, 0.5186]	0.39** [0.3116, 0.4689]
			**Session B**			
ICC	0.33** [0.1568, 0.5618]	0.19** [0.1075, 0.2702]	0.56** [0.4960, 0.6065]	0.07 [-0.0496, 0.2349]	0.24** [0.1352, 0.3588]	0.23** [0.1575, 0.3068]
rho	0.48** [0.4001, 0.5461]	0.37** [0.2869, 0.4509]	0.62** [0.5659, 0.6795]	0.04 [-0.0560, 0.1294]	0.47** [0.3914, 0.5405]	0.40** [0.3145, 0.4729]

Table S17. Similarity (ICC values) of two and five brain states for different clustering strategies in the long, uncentered, 116 ROIs HCP data. A1→B1: Clustering session A1 and back projection on session B1; A1 vs B1: Clustering session A1 and B1 independently.

		State S	State I		State 1	State 2	State 3	State 4	State 5
**a) Clusters based on uncentered data**									
**A1→ B1**	**State S**	0.99	0.67	**State 1**	1.00	0.74	0.74	0.74	0.75
				**State 2**	0.74	0.99	0.57	0.65	0.79
	**State I**	0.65	1.00	**State 3**	0.73	0.54	0.99	0.73	0.42
				**State 4**	0.75	0.65	0.75	0.99	0.64
				**State 5**	0.77	0.79	0.44	0.64	0.99
**A1 vs. B1**	**State S**	0.99	0.68	**State 1**	0.91	0.74	0.82	0.72	0.70
				**State 2**	0.75	0.97	0.62	0.59	0.81
	**State I**	0.64	0.99	**State 3**	0.54	0.52	0.97	0.81	0.48
				**State 4**	0.69	0.61	0.73	0.97	0.78
				**State 5**	0.94	0.81	0.5	0.55	0.86
**b) Clusters based on uncentered data & removing mean of state centroids**									
**A1→ B1**	**State S**	0.92	-0.23	**State 1**	0.96	-0.31	-0.02	-0.46	0.05
				**State 2**	-0.32	0.96	-0.09	-0.49	0.14
	**State I**	-0.20	0.94	**State 3**	-0.02	-0.08	0.95	0.12	-0.16
				**State 4**	-0.41	-0.49	0.15	0.96	-0.27
				**State 5**	0.10	0.09	-0.17	-0.32	0.94
**A1 vs. B1**	**State S**	0.88	-0.23	**State 1**	0.53	-0.16	0.21	-0.36	-0.3
				**State 2**	-0.23	0.87	-0.06	-0.45	0.16
	**State I**	-0.20	0.94	**State 3**	-0.14	-0.07	0.81	0.32	-0.15
				**State 4**	-0.41	-0.58	-0.04	0.86	0.14
				**State 5**	0.66	0.18	-0.13	-0.32	0.22

Table S18. Similarity (cosine distance) of two and five brain states based on different clustering strategies in the long, uncentered, 116 ROIs HCP data. A1→B1: Clustering session A1 and back projection on session B1; A1 vs B1: Clustering session A1 and B1.

		State S	State I		State 1	State 2	State 3	State 4	State 5
**a) Clusters based on uncentered data**									
**A1→ B1**	**State S**	0.00	0.12	**State 1**	0.00	0.11	0.07	0.13	0.14
				**State 2**	0.12	0.00	0.13	0.17	0.14
	**State I**	0.13	0.00	**State 3**	0.07	0.13	0.00	0.10	0.30
				**State 4**	0.12	0.17	0.09	0.00	0.22
				**State 5**	0.13	0.14	0.29	0.22	0.01
**A1 vs. B1**	**State S**	0.01	0.12	**State 1**	0.04	0.11	0.05	0.12	0.18
				**State 2**	0.14	0.01	0.11	0.17	0.12
	**State I**	0.14	0.00	**State 3**	0.19	0.15	0.01	0.06	0.26
				**State 4**	0.17	0.19	0.10	0.01	0.12
				**State 5**	0.04	0.13	0.26	0.26	0.10
**b) Clusters based on uncentered data & removing mean of state centroids**									
**A1→ B1**	**State S**	0.03	1.99	**State 1**	0.04	1.40	0.89	1.49	1.05
				**State 2**	1.40	0.04	1.39	1.51	0.65
	**State I**	1.99	0.02	**State 3**	0.87	1.46	0.01	0.85	1.94
				**State 4**	1.42	1.53	0.80	0.04	1.34
				**State 5**	1.00	0.63	1.92	1.35	0.03
**A1 vs. B1**	**State S**	0.03	1.99	**State 1**	0.49	1.26	0.64	1.30	1.5
				**State 2**	1.05	0.11	1.33	1.62	0.68
	**State I**	1.99	0.02	**State 3**	1.64	1.47	0.04	0.46	1.79
				**State 4**	1.48	1.64	0.95	0.13	0.91
				**State 5**	0.19	0.55	1.85	1.68	0.41

Table S19. Reliability (ICC values) of dFC parameters for different clustering strategies in the long, uncentered, 116 ROIs HCP data. A1→B1: Clustering session A1 and back projection on session B1; A1 vs. B1: Clustering session A1 and B1 individually; ***MDT***: Mean dwell time; ***Prev***: Prevalence; ***ITI***: Inter-transition interval; ***Var***: variability; **S**: segregated state; **I**: integrated state.

	**A1→B1**	**A1 vs. B1**
MDT **S**	**0.30**<br>**[0.0716, 0.5035]**	**0.34**<br>**[0.1494, 0.5166]**
MDT **I**	0.12<br>[-0.0389, 0.3748]	0.09<br>[-0.0364, 0.3660]
Prev **S**	**0.45**<br>**[0.2910, 0.5889]**	**0.44**<br>**[0.2825, 0.5809]**
ITI	0.16<br>[-0.0373, 0.3387]	**0.14**<br>**[0.0152, 0.3556]**
Var **S**	**0.38**<br>**[0.0183, 0.6701]**	**0.34**<br>**[0.0159, 0.6299]**
Var **I**	**0.42**<br>**[0.2798, 0.5304]**	**0.43**<br>**[0.2724, 0.5710]**

Table S20. Reliability (ICC values) of dFC parameters based on five brain states in different clustering strategies in the long, uncentered, 116 ROIs HCP data. A1→B1: Clustering session A1 and back projection on session B1; A1 vs. B1: Clustering session A1 and B1 individually; ***MDT***: Mean dwell time; ***Prev***: Prevalence; ***ITI***: Inter-transition interval; ***Var***: variability; **S**: segregated state; **I**: integrated state. Bold: confidence interval not including zero.

		**A1→B1**	**A1 vs. B1**
MDT	**State 1**	0.06<br>[-0.0938, 0.2101]	-0.06<br>[-0.2052, 0.1162]
	**State 2**	0.11<br>[-0.0199, 0.2576]	0.07<br>[-0.0473, 0.2035]
	**State 3**	**0.21<br>[0.0997, 0.3839]**	**0.27<br>[0.1114, 0.4058]**
	**State 4**	**0.17<br>[0.0302, 0.3166]**	**0.27<br>[0.1172, 0.4162]**
	**State 5**	0.09<br>[-0.0424, 0.2442]	-0.09<br>[-0.2667, 0.1035]
Prev	**State 1**	**0.42<br>[0.2740, 0.5412]**	**0.36<br>[0.2047, 0.5101]**
	**State 2**	**0.40<br>[0.2409, 0.5403]**	**0.34<br>[0.1746, 0.4846]**
	**State 3**	**0.50<br>[0.3637, 0.6092]**	**0.44<br>[0.3031, 0.5544]**
	**State 4**	**0.49<br>[0.3447, 0.6025]**	**0.43<br>[0.2790, 0.5440]**
	**State 5**	**0.40<br>[0.2370, 0.5314]**	0.01<br>[-0.1256, 0.1475]
ITI		0.05<br>[-0.0175, 0.2035]	**0.16<br>[0.0081, 0.3118]**
Var	**State 1**	**0.35<br>[0.1463, 0.5304]**	**0.11<br>[0.0012, 0.2500]**
	**State 2**	0.08<br>[-0.0239, 0.1929]	0.15<br>[-0.0657, 0.3959]
	**State 3**	**0.28<br>[0.1661, 0.3852]**	**0.20<br>[0.0607, 0.3610]**
	**State 4**	**0.28<br>[0.0770, 0.4710]**	**0.22<br>[0.0405, 0.3919]**
	**State 5**	**0.21<br>[0.0494, 0.3736]**	-0.05<br>[-0.1891, 0.1119]

3. Supplementary Discussion

HCP data versus OWN data

The analyses in HCP data confirmed or is consistent with all observations made in OWN data, which are discussed below and were previously reported as a preprint (Fang & Marxen, 2022). This is despite the fact that the datasets underwent different preprocessing and that HCP data has a somewhat lower mean z-value value of 0.28 than OWN data with 0.37 (Fig S9). One reason for this could be the independent component analysis (ICA)-based removal of potential artifacts in HCP data with FSL-FIX (Salimi-Khorshidi et al., 2014), which could have removed noise-driven correlations. However, we observed in another currently unpublished study, when comparing 45 subjects from the UK Biobank, which also employs FSL-FIX, and 45 subjects acquired within a multi-center study (Spanagel et al., 2024) and minimal preprocessing similar to the OWN data, higher correlation values in the ICA-cleaned data. In both cases, the resolution of the fMRI sequences were study-pair-wise the same, i.e., 2mm isotropic in HCP and OWN data, and 2.4 mm isotropic in the UK Biobank and our multi-center study. The multi-band (multi-slice) factor was reduced from 8 to 6 in both cases, respectively. This questions a naive view that ICA-based noise removal surely explains the observed difference in mean z-value. An alternative explanation of the difference could be that the study populations differ indeed in connectivity.

While our previous ICC values in the OWN data are consistent with the results in the HCP data, i.e. demonstrate overlapping confidence intervals, only the HCP data provides reasonable small confidence intervals to compare pipelines and parameters. For example, several high ICC values within the OWN data have confidence intervals that overlap with zero, e.g. MDT_S = 0.62 for the 8’14’’ OWN data and 116 ROIs but has a confidence interval from -0.5 to 0.8. This is a reminder that ICC values that are provided without confidence intervals and are based on small samples can be misleading.

Correlations between dFC parameters

Investigating the correlations between the dFC parameters *Prev*, *MDT*s and *ITI* is important because theoretically different scenarios are possible. While there are certain dependencies between these parameters, any two of them could be independent. For example, if MDT_S increases, $Prev_S$ could decrease if MDT_I increases more strongly than MDT_S. If, however, MDT_S increases while MDT_I decrease, $Prev_S$ also must increase. Similary, if both *MDT*s decrease, *ITI* must decrease, but if *MDT*s are anti-correlated, *ITI* may stay constant.

Conceptually, the picture of brain integration would be most easily interpreted if higher prevalence of one state would be associated with longer *MDT* of the same state and shorter *MDT* of the other state and if *MDT*s would be anti-correlated. In this case, higher levels of integration as measured by *Prev* would be a consequence of a more stable integrated state and a less stable segregated state. We observe this behavior for the centered data. This is reassuring as statements relating to brain integration remain consistent no matter whether they are put in terms of prevalence or MDT_I, i.e. a more integrated brain would have both a higher prevalence and longer MDT_I.

For the centered data, the picture is more complex: *MDTs* correlate positively with each other. This indicates that we only observe that subjects stay longer in one state if they also stay longer in the other, which increase *ITI*. The correlation between *Prev* and *MDT* of the same state is much reduced, especially for the integrated state. Interestingly, *MDT*s are positively correlated with *ITI* even when not centering, which indicates that the negative correlation is not strong enough to keep *ITI* constant.

References

Field Code Changed

Formatted: English (United States)

Diedrichsen, J., Balsters, J. H., Flavell, J., Cussans, E., & Ramnani, N. (2009). A probabilistic MR atlas of the human cerebellum. *Neuroimage, 46*(1), 39-46. doi:10.1016/j.neuroimage.2009.01.045

Esteban, O., Markiewicz, C. J., Blair, R. W., Moodie, C. A., Isik, A. I., Erramuzpe, A., . . . Gorgolewski, K. J. (2019). fMRIPrep: a robust preprocessing pipeline for functional MRI. *Nat Methods, 16*(1), 111-116. doi:10.1038/s41592-018-0235-4

Fang, X., & Marxen, M. (2022). Test-Retest Reliability of Dynamic Functional Connectivity Parameters for a Two-State Model. *bioRxiv*, 2022.2011. 2015.516555.

Glasser, M. F., Sotiropoulos, S. N., Wilson, J. A., Coalson, T. S., Fischl, B., Andersson, J. L., . . . Consortium, W. U.-M. H. (2013). The minimal preprocessing pipelines for the Human Connectome Project. *Neuroimage, 80*, 105-124. doi:10.1016/j.neuroimage.2013.04.127

Leonardi, N., & Van De Ville, D. (2015). On spurious and real fluctuations of dynamic functional connectivity during rest. *Neuroimage, 104*, 430-436. doi:10.1016/j.neuroimage.2014.09.007

Moeller, S., Yacoub, E., Olman, C. A., Auerbach, E., Strupp, J., Harel, N., & Ugurbil, K. (2010). Multiband multislice GE-EPI at 7 tesla, with 16-fold acceleration using partial parallel imaging with application to high spatial and temporal whole-brain fMRI. *Magn Reson Med, 63*(5), 1144-1153. doi:10.1002/mrm.22361

Power, J. D., Barnes, K. A., Snyder, A. Z., Schlaggar, B. L., & Petersen, S. E. (2012). Spurious but systematic correlations in functional connectivity MRI networks arise from subject motion. *Neuroimage, 59*(3), 2142-2154. doi:10.1016/j.neuroimage.2011.10.018

Salimi-Khorshidi, G., Douaud, G., Beckmann, C. F., Glasser, M. F., Griffanti, L., & Smith, S. M. (2014). Automatic denoising of functional MRI data: combining independent component analysis and hierarchical fusion of classifiers. *Neuroimage, 90*, 449-468. doi:10.1016/j.neuroimage.2013.11.046

Schaefer, A., Kong, R., Gordon, E. M., Laumann, T. O., Zuo, X. N., Holmes, A. J., . . . Yeo, B. T. T. (2018). Local-Global Parcellation of the Human Cerebral Cortex from Intrinsic Functional Connectivity MRI. *Cereb Cortex, 28*(9), 3095-3114. doi:10.1093/cercor/bhx179

Shine, J. M., Bissett, P. G., Bell, P. T., Koyejo, O., Balsters, J. H., Gorgolewski, K. J., . . . Poldrack, R. A. (2016). The Dynamics of Functional Brain Networks: Integrated Network States during Cognitive Task Performance. *Neuron, 92*(2), 544-554. doi:10.1016/j.neuron.2016.09.018

Smith, S. M., Beckmann, C. F., Andersson, J., Auerbach, E. J., Bijsterbosch, J., Douaud, G., . . . Consortium, W. U.-M. H. (2013). Resting-state fMRI in the Human Connectome Project. *Neuroimage, 80*, 144-168. doi:10.1016/j.neuroimage.2013.05.039

Spanagel, R., Bach, P., Banaschewski, T., Beck, A., Bermpohl, F., Bernardi, R. E., . . . Heinz, A. (2024). The ReCoDe addiction research consortium: Losing and regaining control over drug intake-Findings and future perspectives. *Addict Biol, 29*(7), e13419. doi:10.1111/adb.13419

Tzourio-Mazoyer, N., Landeau, B., Papathanassiou, D., Crivello, F., Etard, O., Delcroix, N., . . . Joliot, M. (2002). Automated anatomical labeling of activations in SPM using a macroscopic anatomical parcellation of the MNI MRI single-subject brain. *Neuroimage, 15*(1), 273-289. doi:10.1006/nimg.2001.0978

Ugurbil, K., Xu, J., Auerbach, E. J., Moeller, S., Vu, A. T., Duarte-Carvajalino, J. M., . . . Consortium, W. U.-M. H. (2013). Pushing spatial and temporal resolution for functional and diffusion MRI in the Human Connectome Project. *Neuroimage, 80*, 80-104. doi:10.1016/j.neuroimage.2013.05.012

Van Essen, D. C., Smith, S. M., Barch, D. M., Behrens, T. E., Yacoub, E., Ugurbil, K., & Consortium, W. U.-M. H. (2013). The WU-Minn Human Connectome Project: an overview. *Neuroimage, 80*, 62-79. doi:10.1016/j.neuroimage.2013.05.041

Yeo, B. T., Krienen, F. M., Sepulcre, J., Sabuncu, M. R., Lashkari, D., Hollinshead, M., . . . Buckner, R. L. (2011). The organization of the human cerebral cortex estimated by intrinsic functional connectivity. *J Neurophysiol, 106*(3), 1125-1165. doi:10.1152/jn.00338.2011

Zhang, C., Baum, S. A., Adduru, V. R., Biswal, B. B., & Michael, A. M. (2018). Test-retest reliability of dynamic functional connectivity in resting state fMRI. *Neuroimage, 183*, 907-918.
